# Simultaneous augmentation of muscle and bone by locomomimetism through calcium-PGC-1α signaling

**DOI:** 10.1038/s41413-022-00225-w

**Published:** 2022-08-03

**Authors:** Takehito Ono, Ryosuke Denda, Yuta Tsukahara, Takashi Nakamura, Kazuo Okamoto, Hiroshi Takayanagi, Tomoki Nakashima

**Affiliations:** 1 Department of Cell Signaling, Graduate School of Medical and Dental Sciences, Tokyo Medical and Dental University (TMDU), Yushima 1-5-45, Bunkyo-ku, Tokyo, 113-8549 Japan; 2 Department of Orthopaedic Surgery, The Jikei University School of Medicine, 3-25-8 Nishi-Shimbashi, Minato-ku, Tokyo, 105-8461 Japan; 3 School of Dentistry, Tokyo Medical and Dental University (TMDU), Yushima 1-5-45, Bunkyo-ku, Tokyo, 113-8549 Japan; 4 Department of Biochemistry, Tokyo Dental College, Kandamisakicho 2-9-18, Chiyoda-ku, Tokyo, 101-0061 Japan; 5 Department of Osteoimmunology, Graduate School of Medicine and Faculty of Medicine, The University of Tokyo, Hongo 7-3-1, Bunkyo-ku, Tokyo, 113-0033 Japan; 6 Department of Immunology, Graduate School of Medicine and Faculty of Medicine, The University of Tokyo, Hongo 7-3-1, Bunkyo-ku, Tokyo, 113-0033 Japan

**Keywords:** Bone, Homeostasis

## Abstract

Impaired locomotion has been extensively studied worldwide because those afflicted with it have a potential risk of becoming bedridden. Physical exercise at times can be an effective remedy for frailty, but exercise therapy cannot be applied in all clinical cases. Medication is safer than exercise, but there are no drugs that reinforce both muscle and bone when administered alone. Multiple medications increase the risk of adverse events; thus, there is a need for individual drugs targeting both tissues. To this end, we established a novel sequential drug screening system and identified an aminoindazole derivative, locamidazole (LAMZ), which promotes both myogenesis and osteoblastogenesis while suppressing osteoclastogenesis. Administration of this drug enhanced locomotor function, with muscle and bone significantly strengthened. Mechanistically, LAMZ induced Mef2c and PGC-1α in a calcium signaling–dependent manner. As this signaling is activated upon physical exercise, LAMZ mimics physical exercise. Thus, LAMZ is a promising therapeutic drug for locomotor diseases, including sarcopenia and osteoporosis.

## Introduction

The modern lifestyle in developed countries has emancipated us from heavy labor but has also resulted in a pattern of insufficient physical activity.^1,2^ Physical inactivity causes frailty of the muscle and bone (i.e., sarcopenia and osteoporosis), which can result in people becoming bedridden. In addition, physical inactivity is associated with other diseases, including cardiovascular disease, stroke and diabetes, making it one of the major risk factors for death.^3^ The socioeconomic burden of treating these diseases is growing progressively, so considerable attention is being paid to increasing physical activity.^4^

Despite this, exercise is not appropriate for all patients because of their specific conditions, such as cerebrovascular disease, dementia or when a patient has already become bedridden. In such cases, drug therapy would be an efficacious solution. Several clinical trials are underway to develop therapeutic drugs for sarcopenia, targeting androgen receptor as well as myostatin,^5,6^ but there have not been any drugs yet approved for the improvement of the muscle loss that occurs in sarcopenia.^7^ There are a variety of drugs for the treatment of bone loss: vitamin D_3_, teriparatide, selective estrogen receptor modulators (SERMs), bisphosphonates, denosumab and romosozumab.^8,9^ Although sarcopenia and osteoporosis often coexist,^10,11^ most of these drugs exert effects only on bone. Therefore, patients might be compromised by multiple medication regimens, even if a drug for sarcopenia were developed. Furthermore, elderly patients often have other diseases and thus would be prescribed additional medications. The prescription of multiple drugs, polypharmacy, is a common cause of poor adherence or noncompliance and drug interactions that often result in adverse events. From the viewpoint of avoiding polypharmacy, these diseases of frailty should be treated with a single drug targeting both conditions.

## Results

### Sequential screening identified locamidazole (LAMZ) as a candidate therapeutic drug for impaired locomotion

**Fig. 1:**
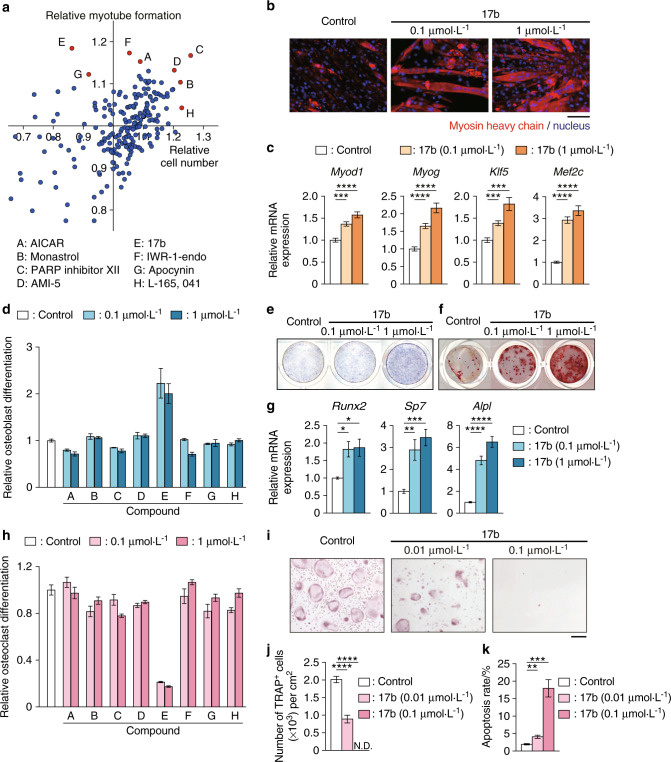
Sequential screening identified 17b (locamidazole: LAMZ) as a candidate therapeutic drug for impaired locomotion. **a** Primary screening for extracting compounds that enhance the proliferation and/or differentiation of myocytes. The relative proliferation and myotube formation are plotted in the scattergram. Each dot represents the corresponding compound. Compounds A–H are as follows: A, AICAR; B, monastrol; C, PARP inhibitor XII; D, AMI-5; E, 17b; F, IWR-1-endo; G, apocynin; and H, L-165,041. The dots depicted in red underwent further screening. **b** Representative immunocytofluorescence images of C2C12 cells. Myosin heavy chain (red); nuclei (blue). **c** mRNA expression of myogenic genes. **d** Secondary screening for extracted compounds enhancing osteoblastogenesis based on alkaline phosphatase (ALP) activity. **e** Representative ALP staining images of osteoblasts. **f** Representative images of the mineralization of osteoblasts. **g** mRNA expression of osteoblastic genes. **h** Tertiary screening for extracting compounds that suppress osteoclastogenesis based on *Ctsk* expression indicated by LacZ activity. **i** Representative images of tartrate–resistant acid phosphatase (TRAP) staining. **j** Number of TRAP^+^ multinucleated cells. N.D. not detected. **k** Apoptosis ratio of osteoclasts detected by TdT-mediated dUTP nick-end labeling (TUNEL) assay. The data of the effects of 17b were obtained from 3 independent experiments with replicates of three wells. Scale bar, 100 μm. Statistical analyses were carried out using one-way ANOVA followed by Dunnett’s multiple-comparison test or Brown–Forsythe ANOVA test and Dunnett’s T3 test. The error bars show the mean ± s.e.m. *P < 0.05; **P < 0.01; ***P < 0.001; ****P < 0.000 1

Reinforcement of the muscle and bone during exercise coincides with anabolic changes in both muscle and bone (e.g., increase in proliferation and/or differentiation of myocytes and osteoblasts and a decrease of these in osteoclasts). In search of drugs that can be a substitute for exercise, we established a sequential drug screening system, in which the differentiation of three lineages of cells in muscle and bone (myocytes, osteoblasts and osteoclasts) was quantified. First, we developed a novel method for quantifying the proliferation and differentiation of myocytes. Among 296 compounds in a chemical library, eight were found to enhance the proliferation and/or differentiation of C2C12 cells, a myocyte cell line (Fig. 1a). Compound 17b, an aminoindazole derivative that showed the strongest promotion of myotube formation, enhanced myosin heavy chain expression (Fig. 1b). This compound increased the expression of *Myod1*, *Myog*, *Klf5* and *Mef2c*, transcription factors known to be induced during myogenesis and to increase differentiation (Fig. 1c).^12,13^

The eight compounds were examined for their effects on osteoblastogenesis using MC3T3-E1, an osteoblastic cell line. A cell culture-based protocol for quantifying the activity of alkaline phosphatase (ALP), an osteoblast marker, is commonly used.^14,15^ This protocol consists of multiple steps, through which artifacts can emerge, so we devised a simplification that reduces the technical burden and prevents the technical errors associated with such multiple steps (see methods) (Fig. S1a). Using this system, 17b was demonstrated to have the most potent capacity to enhance osteoblastogenesis (Fig. 1d), a result that was confirmed by ALP staining (Fig. S1b). We found that 17b enhanced ALP activity, mineralization and the expression of the osteogenic genes *Runx2*, *Sp7* (Osterix) and *Alpl* (alkaline phosphatase) in calvarial cells from neonatal mice (Fig. 1e–g).

The eight candidates were further evaluated for their effects on osteoclastogenesis based on the activity of an osteoclastic enzyme, cathepsin K (CtsK).^16^ Genetically modified mice expressing β-galactosidase (LacZ) in a *Ctsk* promoter-dependent manner figure legend of Fig. 4 for the quantification of CtsK activity (Fig. S1c, d).^17,18^ Among the candidates, 17b was revealed to most efficiently suppress osteoclastogenesis (Fig. 1h–j). Osteoclast apoptosis was enhanced by this compound, with cleaved caspases and proapoptotic genes increased (Fig. 1k and Fig. S1e, f). Thus, Compound 17b enhanced both myogenesis and osteoblastogenesis while suppressing osteoclastogenesis via direct effects. Therefore, this compound was called “locamidazole”, after “locomotor” and the chemical backbone “aminoindazole”, LAMZ in short.

### Oral administration of LAMZ augments both muscle and bone

**Fig. 2:**
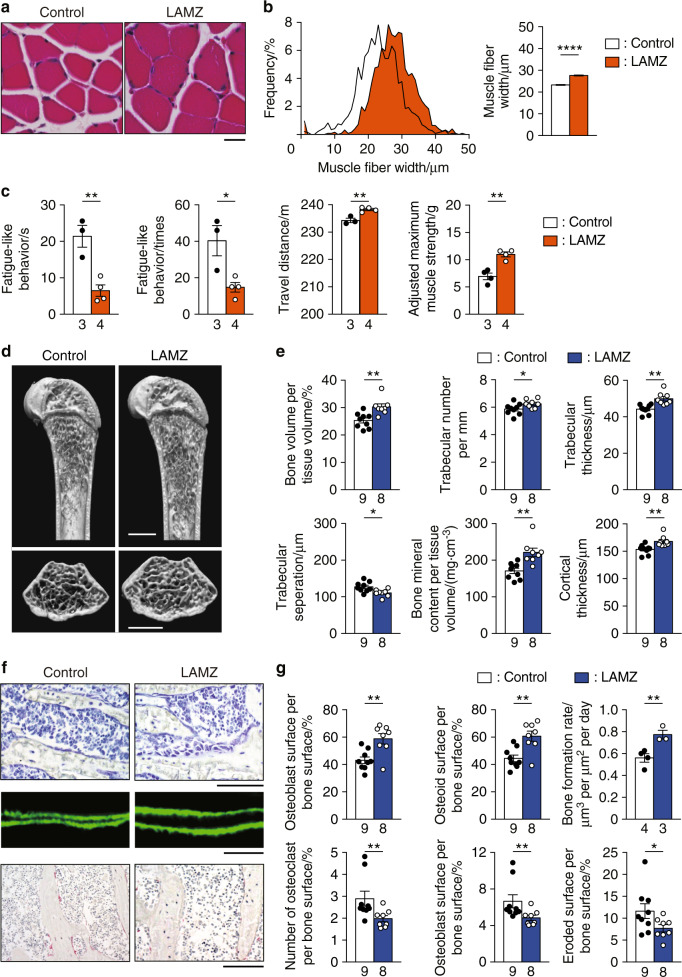
Oral administration of LAMZ augments both muscle and bone. **a** Representative histological images of the soleus muscle of mice orally treated with LAMZ or a control emulsion. Cross sections were stained with hematoxylin and eosin. Scale bar, 20 μm. **b** Distribution and mean value of the fiber width of the soleus muscle. Four sections per mouse and four mice in each group were analyzed. In total, the numbers of fibers measured were 1 456 and 1 262, respectively. **c** Locomotor functions of mice orally administered LAMZ. **d** Representative microcomputed tomography (CT) images of the femurs of the mice orally treated with LAMZ or the control emulsion. Upper, sagittal section; lower, transverse section of the metaphyseal area. Scale bar, 1 mm. **e** Bone parameters obtained by micro-CT analyses. **f** Bone morphometric analysis of the proximal tibia of the mice orally treated with LAMZ or control emulsion. Upper, toluidine blue staining (scale bar, 100 μm); middle, calcein labeling (scale bar, 20 μm); lower, tartrate–resistant acid phosphatase (TRAP) staining (scale bar, 100 μm). **g** Parameters of osteoblastic bone formation and osteoclastic bone resorption. Statistical analyses were carried out using Student’s *t* test. The number of biological replicates is described below each bar. The error bars show the mean ± s.e.m. *P < 0.05; **P < 0.01; ****P < 0.000 1

The in vitro efficacy of LAMZ prompted us to conduct in vivo analyses. LAMZ was transferred to the blood when administered orally and thus was administered once daily for 14 days; this treatment did not affect body weight (Fig. S2a–c). At the endpoint of the experiment, the drug was detected in the blood, muscle and bone, with no detectable adverse effects on hematologic parameters (Fig. S2d–f). There were no apparent abnormal findings that would indicate possible side effects (Fig. S2g–j). The muscle fiber width was larger in the mice treated with LAMZ than in the control mice, with no obvious manifestation of muscle/tendon degeneration (Fig. 2a, b and Fig. S3a–c). The LAMZ-treated mice ran on a treadmill running machine with fewer episodes of fatigue-like behavior (Fig. 2c). The travel distance was significantly increased by LAMZ, although it was a small increase (Fig. 2c). The maximal muscle strength of these mice was greater as well (Fig. 2c). Microcomputed tomography (micro-CT) analysis indicated that the trabecular and cortical bone parameters were significantly higher in the LAMZ-treated mice (Fig. 2d, e). More osteoblasts were observed, with a higher rate of bone formation, while there were fewer osteoclasts in the LAMZ-treated mice, with lower bone resorption activity (Fig. 2f, g). Although Sclerostin (*Sost*) was found to increase as osteoblastogenesis was enhanced by LAMZ, there was no significant increase in its expression in cultured osteocytes or in bone tissue (Fig. S3d). LAMZ did not induce degeneration of the articular cartilage (Fig. S3e, f). These results demonstrated that LAMZ reinforces both muscle and bone in vivo without any obvious adverse effects on other tissues.

The LAMZ analog linifanib (Fig. S4a) was examined for its effects on muscle and bone. This drug enhanced myogenesis and osteoblastogenesis while suppressing osteoclastogenesis in vitro, similar to LAMZ (Fig. S4b–d). Oral administration of linifanib resulted in a significant increase in muscle fiber width (Fig. S4e, f). There was also an increase in bone mass and mineral density (Fig. S4g, h), indicating that aminoindazole derivatives share anabolic effects on muscle and bone.

### PGC-1α mediates the anabolic effects of LAMZ on muscle and bone

**Fig. 3:**
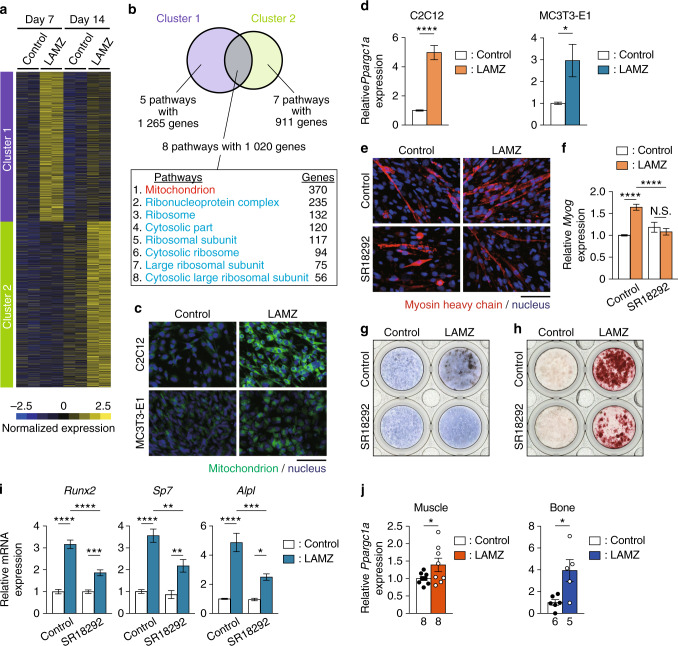
PGC-1α mediates the anabolic effects of LAMZ on muscle and bone. **a** Gene clustering analysis based on comprehensive gene expression profiles of LAMZ-treated cells. The genes upregulated by LAMZ were clustered. **b** Venn diagram indicating the pathways commonly enriched in the clusters. The numbers of pathways and genes are described. **c** Mitochondrial content in the cells treated with LAMZ. Mitochondria (green); nuclei (blue). **d** mRNA expression of *Ppargc1a* (PGC-1α) in the LAMZ-treated cells. **e** Representative immunocytofluorescence images of the C2C12 cells treated with the PGC-1α inhibitor SR18292 together with LAMZ. Myosin heavy chain (red); nuclei (blue). **f** mRNA expression of *Myog*. **g** Representative ALP staining images of the MC3T3-E1 cells treated with SR18292 together with LAMZ. **h** Representative Alizarin Red S staining images of MC3T3-E1 cells. **i** mRNA expression of osteoblastic genes. **j** In vivo expression of *Ppargc1a* in the tissues of the mice orally administered LAMZ. In vitro experiments were repeated three times with replicates of 2 or 3 wells. Scale bar, 100 μm. For the comparison of 2 groups, statistical analyses were carried out using Welch’s *t* test. For multiple comparisons, two–way ANOVA and Tukey’s multiple-comparison test were applied. The number of biological replicates is described below each bar. The error bars show the mean ± s.e.m. *P < 0.05; **P < 0.01; ***P < 0.001; ****P < 0.000 1; N.S. not significant

To investigate the functional mode by which LAMZ augments muscle and bone, we conducted RNA sequencing and Gene Ontology analyses. These analyses identified the genes highly expressed in the LAMZ-treated cells and the pathway enriched in these cells (Fig. 3a and Fig. S5). Mitochondrial genes were enriched in the LAMZ-treated cells, with the largest number of genes involved (Fig. 3b). Indeed, LAMZ treatment increased the mitochondrial content in both myocytes and osteoblasts (Fig. 3c).

The transcriptional coactivator PGC-1α is known to enhance mitochondrial biogenesis. This coactivator is also known for its maintenance of muscle and bone, acting cooperatively with transcription factors.^19–21^ The mRNA of PGC-1α (*Ppargc1a*) was highly expressed in the myocytes and osteoblasts treated with LAMZ (Fig. 3d). The PGC-1α inhibitor SR18292 abrogated the acceleration of myotube formation and the expression of *Myog* induced by LAMZ (Fig. 3e, f). In osteoblastic cells, PGC-1α inhibition blunted the acceleration of osteoblastogenesis, mineralization and the expression of osteoblastic genes by LAMZ (Fig. 3g–i). These results indicate that LAMZ exerts effects on myotube and osteoblast differentiation via PGC-1α. Oral administration of LAMZ increased the expression of *Ppargc1a* in muscle and bone in vivo (Fig. 3j). We found that the myogenesis of human myoblasts stimulated with LAMZ was enhanced along with the upregulated expression of *PPARGC1A* (Fig. S6a, b). Osteoblastic differentiation and mineralization of human mesenchymal stem cells (MSCs) were promoted by LAMZ with increased expression of *PPARGC1A* (Fig. S6c, d).

To determine whether LAMZ functions via PGC-1α, we injected SR18292 into mice at the same time as oral administration of LAMZ (Fig. S7a). Inhibition of PGC-1α by SR18292 diminished the increase in muscle fiber width (Fig. S7b, c), abolishing the enhancement of muscular strength (Fig. S7d). PGC-1α inhibition was also found to blunt the effects of LAMZ on the bone (Fig. S7e, f). Together, these results indicate that the effects of LAMZ on muscle and bone are mediated by PGC-1α.

### LAMZ induces PGC-1α through calcium signaling

**Fig. 4:**
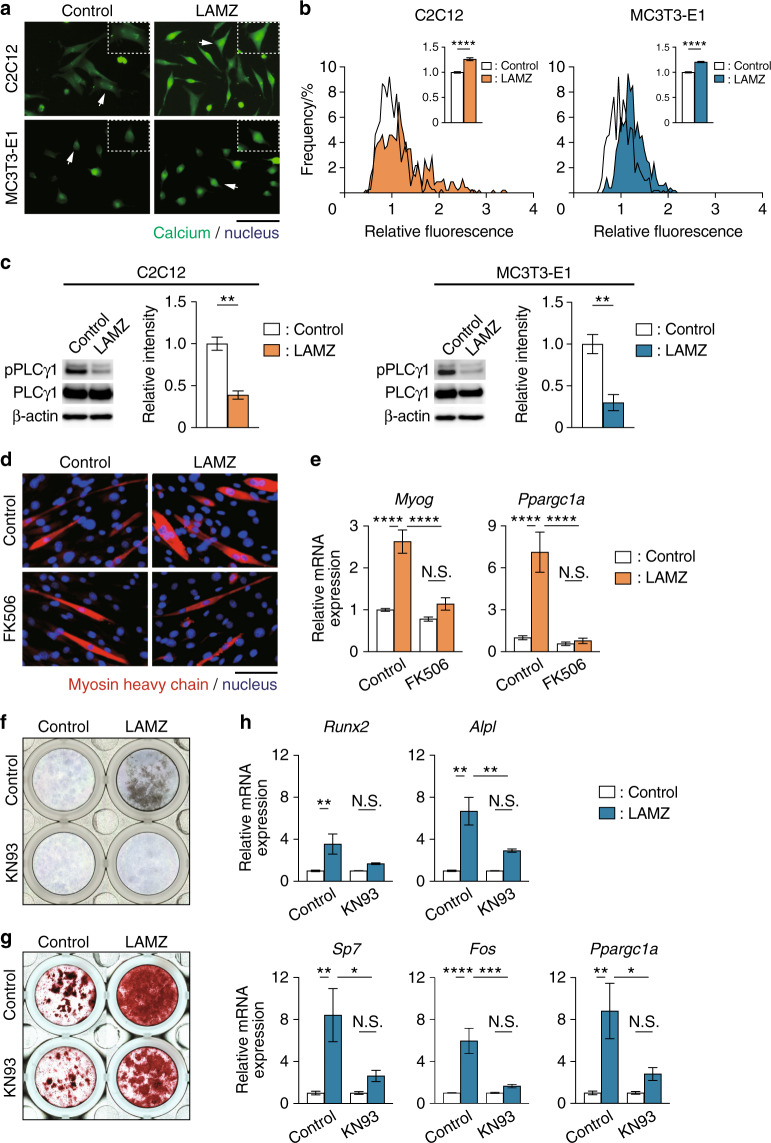
LAMZ induces calcium signaling to enhance myogenesis and osteoblastogenesis. **a** Representative images of intracellular calcium in C2C12 cells and MC3T3-E1 cells treated with LAMZ. Calcium (green); nuclei (blue). Scale bar, 50 μm. **b** Distribution and mean value (inset) of the fluorescence intensity that indicates the intracellular calcium. In total, the numbers of cells measured were 369 (control C2C12 cells), 457 (LAMZ-treated C2C12 cells), 499 (control MC3T3-E1 cells) and 507 (LAMZ-treated MC3T3-E1 cells). **c** Western blotting analysis and subsequent densitometric quantitation of the inhibitory phosphorylation of PLCγ1 in C2C12 cells and MC3T3-E1 cells. **d** Representative immunocytofluorescence images of C2C12 cells treated with the calcineurin inhibitor FK506 together with LAMZ. Myosin heavy chain (red); nuclei (blue). Scale bar, 100 μm. **e** mRNA expression of *Myog* and *Ppargc1a*. **f** Representative ALP staining images of the MC3T3-E1 cells treated with LAMZ and KN93, an inhibitor of calcium signaling. **g** Representative Alizarin Red S staining images of MC3T3-E1 cells. **h** mRNA expression of the osteoblastic genes, *Fos* and *Ppargc1a*. Experiments were repeated three times with replicates of 2 or 3 wells. Scale bar, 100 μm. For the comparison of two groups, statistical analyses were carried out using Student’s *t* test or Welch’s *t* test. For multiple comparisons, two–way ANOVA and Tukey’s multiple-comparison test were applied. The error bars show the mean ± s.e.m. *P < 0.05; **P < 0.01; ****P < 0.000 1; N.S. not significant

Exercise induces Ca^2+^ influx into the cytosol, eliciting the calcium signaling pathway, which results in PGC-1α expression.^22^ LAMZ was shown to increase intracellular Ca^2+^ in both myocytes and osteoblasts (Fig. 4a, b), with a decrease in the inhibitory phosphorylation of PLCγ1,^23^ a transducer of calcium signaling (Fig. 4c). We thus asked if LAMZ increases PGC-1α by facilitating this pathway. Blockade of calcium signaling by the addition of the calcineurin inhibitor FK506 attenuated the enhancement of myogenesis by LAMZ (Fig. 4d, e). The treatment also reversed the upregulation of *Ppargc1a* expression induced by LAMZ (Fig. 4e). Administration of KN93, an inhibitor of a component molecule of calcium signaling, CaMKII, blunted the acceleration of osteoblastogenesis and mineralization induced by LAMZ (Fig. 4f–h). The increase in osteoblastic genes and the calcium-responsive gene *Fos* by LAMZ was diminished by KN93 (Fig. 4h). Furthermore, *Ppargc1a* induced by LAMZ was attenuated (Fig. 4h). Thus, LAMZ targets the calcium signaling pathway to stimulate myogenesis and osteogenesis.

**Fig. 5:**
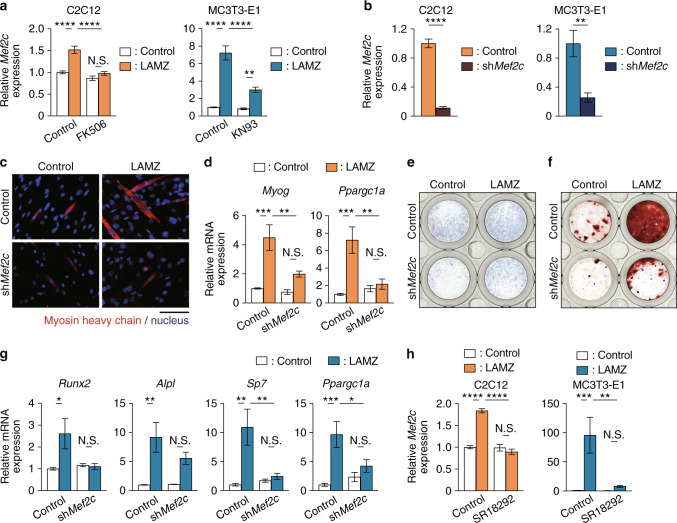
LAMZ induces Mef2c to enhance myogenesis and osteoblastogenesis. **a** mRNA expression of *Mef2c* in the C2C12 cells and MC3T3-E1 cells treated with calcium signal inhibitors together with LAMZ. **b** mRNA expression of *Mef2c* in the C2C12 cells and MC3T3-E1 cells infected with lentiviruses encoding sh*Mef2c*. **c** Representative immunocytofluorescence images of the C2C12 cells infected with lentiviruses encoding sh*Mef2c* and treated with LAMZ. Myosin heavy chain (red); nuclei (blue). Scale bar, 100 μm. **d** mRNA expression of *Myog* and *Ppargc1a*. **e** Representative ALP staining images of the MC3T3-E1 cells infected with lentiviruses encoding sh*Mef2c* and treated with LAMZ. **f** Representative images of Alizarin Red S staining of MC3T3-E1 cells. **g** mRNA expression of osteoblastic genes and *Ppargc1a*. **h** mRNA expression of *Mef2c* in the C2C12 cells and MC3T3-E1 cells treated with the PGC-1α inhibitor SR18292. Experiments were repeated 3 times with replicates of two wells. For the comparison of two groups, statistical analyses were carried out using Welch’s t test. For multiple comparisons, two–way ANOVA and Tukey’s multiple-comparison test were applied. The error bars show the mean ± s.e.m. *P < 0.05; **P < 0.01; ***P < 0.001; ****P < 0.000 1; N.S. not significant

The transcription factor Mef2c functions as a molecular hub in calcium signaling, leading to the expression of various genes, including *Ppargc1a* and *Mef2c* itself.^24–26^ Mef2c was shown to be induced both in myocytes and osteoblasts stimulated with LAMZ but not when calcium signaling was inhibited (Fig. 5a). *Mef2c* was knocked down using shRNA to examine whether this transcription factor mediates the effects of LAMZ (Fig. 5b). The myocytes that underwent *Mef2c* knockdown were unresponsive to LAMZ: the expression of the myosin heavy chain, *Myog* and *Ppargc1a* was not upregulated by LAMZ in these cells (Fig. 5c, d). In osteoblasts with knockdown of *Mef2c*, there was no significant acceleration in osteoblastic differentiation and mineralization by LAMZ (Fig. 5e, f). The expression of osteoblastic genes and *Ppargc1a* was not significantly increased by LAMZ when *Mef2c* was knocked down (Fig. 5g). Interestingly, *Mef2c* upregulation by LAMZ was abolished by SR18292 both in myocytes and osteoblasts (Fig. 5h), suggesting reciprocal regulation between Mef2c and PGC-1α. Taken together, these results indicate that LAMZ facilitates the calcium signaling pathway to induce Mef2c and PGC-1α, thus enhancing myogenesis and osteogenesis.

### LAMZ ameliorates the frailty of muscle and bone

**Fig. 6:**
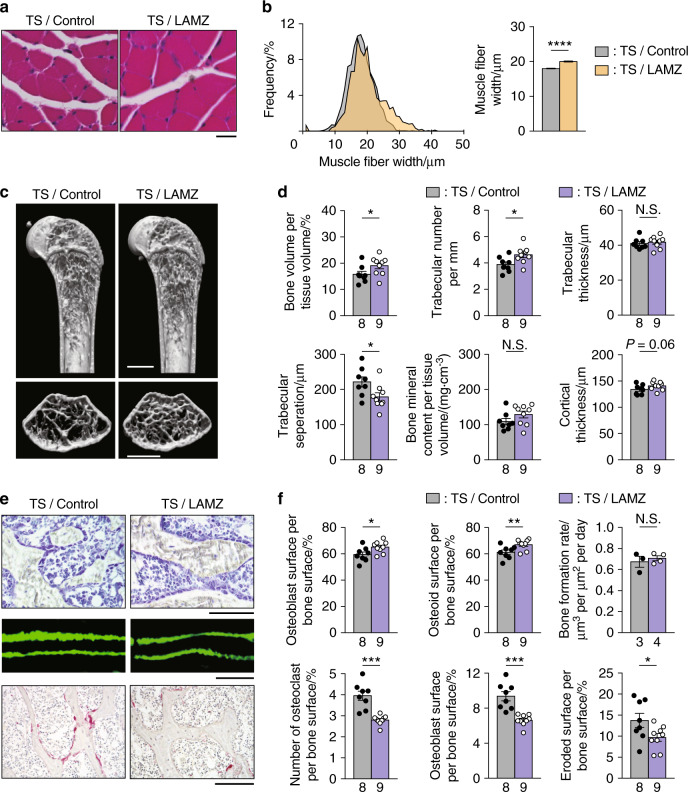
Oral administration of LAMZ ameliorates the frailty of muscle and bone. **a** Representative histological images of the soleus muscle of tail suspension (TS) model mice orally treated with LAMZ or a control emulsion. Cross sections of the muscle were stained with hematoxylin and eosin. Scale bar, 20 μm. **b** The distribution and mean value of the fiber width of the soleus muscle. Four sections per mouse and four mice in each group were analyzed. In total, the numbers of fibers measured were 1963 and 1530. **c** Representative microcomputed tomography (CT) images of the femur of the disuse model mice orally treated with LAMZ or the control emulsion. Upper, sagittal section; and lower, transverse section of the metaphyseal area. Scale bar, 1 mm. **d** Bone parameters obtained by micro-CT analyses. **e** Bone morphometric analysis of the proximal tibia of the mice orally treated with LAMZ or a control emulsion. Upper, toluidine blue staining (scale bar, 100 μm); middle, calcein labeling (scale bar, 20 μm); and lower, tartrate–resistant acid phosphatase (TRAP) staining (scale bar, 100 μm). **f** Parameters of osteoblastic bone formation and osteoclastic bone resorption. Statistical analyses were carried out using Student’s *t* test or Welch’s *t* test. The number of biological replicates is described below each bar. The error bars show the mean ± s.e.m. *P < 0.05; **P < 0.01; ***P < 0.001; ****P < 0.000 1; N.S. not significant

To determine whether LAMZ has efficacy as a novel therapeutic drug for impaired locomotion, we orally administered the drug to tail suspension model mice, a disuse model associated with unloading (Fig. S8a), in which both the muscle and bone of the hindlimb become frail.^27^ Oral administration of LAMZ significantly upregulated *Ppargc1a* expression in muscle and bone (Fig. S8b). In mice treated with this drug during tail suspension, there was an increase in the width of the muscle fibers (Fig. 6a, b). Micro-CT analysis revealed that LAMZ treatment increased bone mass (Fig. 6c, d). A significant increase in osteoblasts and the osteoid surface was observed in these mice (Fig. 6e, f). There was no significant difference in the bone formation rate, owing to the increase in bone formation by tail suspension itself (Fig. 2f, g and Fig. 6e, f). Osteoclastic bone resorption was decreased by LAMZ treatment in these mice (Fig. 6e, f). The reduction of the muscle and bone induced by the disuse model was mitigated by LAMZ treatment (Fig. S8c–e).

**Fig. 7:**
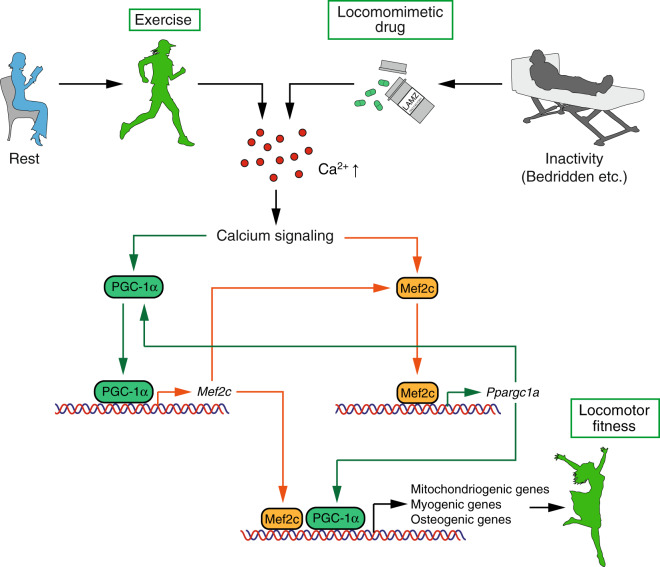
LAMZ augments muscle and bone by mimicking calcium signaling to induce PGC-1α. Schematic of LAMZ function in the augmentation of muscle and bone. Exercise increases the calcium concentration in muscle and bone cells, inducing calcium signaling. Under this signaling, PGC-1α and Mef2c are activated and reciprocally drive their expression. Mitochondriogenic, myogenic and osteogenic genes are induced as well, together augmenting the muscle and bone. Thus, LAMZ facilitates the calcium signaling pathway and restores locomotor fitness

Although LAMZ was demonstrated to have therapeutic effects on both muscle and bone, it may not be useful in patients who are unable to take medicine orally due to medical conditions such as a lack of consciousness. To make LAMZ available to such patients, we also conducted a subcutaneous injection study of LAMZ (Fig. S9a). Given that the AUC was approximately half of that of oral administration, the injection was performed twice daily (Fig. S9b). The injection resulted in the enlargement of muscle fibers (Fig. S9c, d), as well as improvement of bone mass and mineral density (Fig. S9e, f). Thus, LAMZ functions as a therapeutic drug for impaired locomotion by reinvigorating muscle and bone via PGC-1α, mimicking physical exercise (Fig. 7). The availability of multiple routes of administration should prove useful in patients with certain limitations.

## Discussion

Physical exercise is known to be beneficial for improving the function of locomotor tissues, as well as the metabolic, circulatory and neuronal systems. Nevertheless, exercise therapy for reinvigorating muscle and bone cannot be applied in all clinical cases, and drug therapy is an attractive alternative. However, there is no single drug that is effective for both sarcopenia and osteoporosis when administered alone. This issue is partly because the drugs for musculoskeletal diseases have been developed based on the discovery of functional molecules in myotubes, osteoblasts or osteoclasts. Screening systems based on phenotypic output, instead of functional molecules, is a direct solution to allow the discovery of drugs that have the requisite functions in multiple cell types, thus saving on both cost and clinical development time, notorious bottlenecks in drug development.^28^ Based on this idea, we have developed phenotype–based screening systems for these three lineages of cells (Fig. 1a, d, h) and identified a locomomimetic drug LAMZ, which was not previously reported to have functional effects on muscle and/or bone.

Calcium signaling is a major response to mechanical stimulation, including physical exercise.^29^ Upon stimulation, there is an influx of Ca^2+^ into the cytosol of muscle and bone cells, which further activates downstream molecules. Calcineurin is a calmodulin-dependent serine/threonine protein phosphatase that dephosphorylates various proteins in many biological processes.^30^ CaMKII is another calmodulin-dependent serine/threonine protein phosphatase, the substrates of which also encompass a wide range of proteins.^31^ Both of these phosphatases activate Mef2c, and it in turn promotes the expression of Ca^2+^-regulated genes, including *Ppargc1a* and *Mef2c* itself.^24,32,33^ LAMZ was shown to replicate the calcium signaling pathway by stimulating gene expression through calcineurin and CaMKII.

Mef2c is a transcription factor, the activity of which is provoked upon calcium signaling. Its expression is known to be broad, thus regulating various biological processes, including the nervous, immune and musculoskeletal systems.^34^ In the process of skeletal muscle development, Mef2c cooperates with other transcription factors to prompt myogenesis and regeneration.^35,36^ It has also been shown that this transcription factor is involved in mineralization of the bone.^37–40^ Furthermore, Mef2 family molecules have been reported to increase mitochondrial content in several types of cells, indicating that LAMZ increases mitochondrial content in the muscle and bone via Mef2c.^33,41,42^ One of the major targets of Mef2c is PGC-1α, which plays critical roles in many biological processes, including lipid, glucose, muscle and bone metabolism.^25,43,44^ In skeletal muscle, it was reported that physical exercise induces PGC-1α, increasing muscle volume and exercise tolerance, with an increase in mitochondria.^19,45^ In bone, PGC-1α skews the fate of skeletal stem cells toward osteoblasts and enhances mineralization, again with an increase in mitochondrial content.^20^ These effects induced by Mef2c and PGC-1α in the muscle and bone were replicated by LAMZ treatment. Thus, LAMZ mimics calcium signaling, which can be driven by physical exercise, to stimulate Mef2c and PGC-1α expression, resulting in the augmentation of muscle and bone (Fig. 7).

Our results indicate that LAMZ is transferred to the blood after both oral and subcutaneous administration (Fig. S2b and 9b), suggesting that it can distribute to other organs, including the liver, fat and brain, enhancing PGC-1α expression and improving the functions of these organs. Recently, it was reported that PGC-1α has a protective effect on aging by maintaining mitochondrial homeostasis,^46^ suggesting a possible application of LAMZ to sedentary diseases and metabolic disorders.

In conclusion, we have established a novel, three–part drug screening system and discovered a locomomimetic drug, LAMZ. LAMZ enhances myogenesis and osteoblastogenesis via stimulation of calcium-PGC-1α signaling. It can be administered by multiple routes and improves both muscle and bone mass, resulting in increased capacity for locomotion. Therefore, LAMZ has potential as a therapeutic drug for patients with impaired locomotion, such as those with sarcopenia and osteoporosis.

## Materials and methods

### Experimental animals

Six-week-old C57BL/6 J male mice were purchased from Clea Japan and underwent acclimatization for a week before experiments. The CAG–CAT–*lacZ*^Tg/Tg^ mouse was kindly provided by J. Miyazaki.^17^ The *Ctsk*^*Cre*/Wt^ mouse was generated as previously described.^18^ These mice were maintained at Tokyo Medical and Dental University under specific pathogen-free conditions. During the course of the experiment, the body weight of the mice was measured every day. The number of mice used in each experiment is described in the corresponding figure. All animal experiments were approved by the Institutional Animal Care and Use Committee and Genetically Modified Organisms Safety Committee of Tokyo Medical and Dental University (approval No. A2021-028C2 and G2018-028C15, respectively) and conducted in accordance with the guidelines concerning the management and handling of experimental animals.

### Drug administration

Mice were orally administered 10 mg·kg^−1^ per day of LAMZ, linifanib or a control emulsion once a day for 14 days. Oral administration was performed using a feeding needle (Natsume Seisakusho).^47^ The LAMZ and linifanib emulsions were prepared to be 1 mg·mL^−1^ in the following solution: 2.5% ethanol, 2.5% dimethyl sulfoxide (DMSO), 5% Tween 80 and 25% PEG 400 in phosphate–buffered saline (PBS).^48^ For subcutaneous injection, LAMZ was dissolved into propylene glycol at a concentration of 1.2 mg·mL^−1^. Five mL·kg^−1^ of the solution was injected into the backs of the mice twice daily for 14 days. The SR18292 solution for the intraperitoneal injection was prepared to be 6 mg·mL^−1^ in the following solution: 10% DMSO and 10% Tween 80 in PBS.^49^ A total of 7.5 mL·kg^−1^ of the solution was injected per day for 14 days.

### Mouse models

To mimic disuse–induced frailty, we performed a tail-suspension procedure on the mice with a suspension device (Takatsuka Life Science). The tail of the subject mouse was hung from the beam of the device using a chain and an adhesive bandage so the hindlimb of the subject did not reach the floor. Endurance capacity was tested using a treadmill device (Muromachi Kikai). Mice underwent forced running on the device with the following parameters: inclination, 7°; speed, 16 m·min.^−1^; duration, 15 min; electric shock intensity, 2 mA. The contact time and frequency on the posterior edge of the belt were automatically recorded. Fatigue-like behavior (times) was defined as the number of contacts in the last 10 min. Fatigue-like behavior (sec) was calculated as the time of contact in the last 10 min. The distance traveled was acquired by multiplying the speed and running time without fatigue-like behavior in the last 10 min. Maximum muscle strength was measured using a grip strength meter (Muromachi Kikai). A subject mouse was placed on a wire mesh, and its tail was gently pulled. The resistance strength against being pulled away from the mesh was measured 10 times per mouse. The maximum and minimum values were excluded, and the mean value of the remaining values was calculated.

### Peripheral blood collection

Peripheral blood was collected from the submandibular vein or the heart. For the analysis of the LAMZ concentration in the plasma, blood was collected in a microtube containing EDTA, the final concentration of which was 2 mg·mL^−1^. Samples then underwent centrifugation (1 600 g, 10 min, 4 °C), and plasma was collected in microtubes. The plasma was snap-frozen using liquid nitrogen and stored at –80 °C. For the blood cell count, blood flowing from the mandible was collected using a hematocrit capillary treated with heparin (AS ONE) and immediately transferred into microtubes containing EDTA at a final concentration of 2 mg·mL^−1^. Blood cells were counted using a diagnostic device (Nihon Kohden). Blood glucose was measured after fasting for 14 hours using a diagnostic device (Nipro). For serum biochemistry, peripheral blood was collected from the heart. After collection, the blood sample was left in a microtube at room temperature for 30 min. Then, the sample underwent centrifugation (10 000 r·min^−1^1, 10 min., RT), and the serum was collected in another microtube and stored at –80 °C. The serum biochemistry profile was analyzed at Oriental Yeast Co., Ltd.

### Tissue harvest and preparation

For the evaluation of the bone formation rate, mice were injected with calcein (16 mg·kg^−1^) 4 days and 1 day prior to sacrifice. After the mice were sacrificed by anesthesia, the bone samples for the micro-CT analysis and histomorphometric analysis were fixed with 70% ethanol at 4 °C. The samples for histological analyses underwent fixation with 4% paraformaldehyde (PFA) at 4 °C overnight. The tissues for the gene expression analyses were minced in Sepasol^®^-RNA I Super G (Nacalai Tesque) and stored at –80 °C. The muscle and bone samples for measuring the drug concentration were snap-frozen using liquid nitrogen and stored at –80 °C.

### Quantification of the drug concentration

Plasma, muscle and bone were mixed with methanol and an internal control. These samples were processed with 0.1 mol·L^−1^ NaOH, a solid-phase extraction plate (Waters) and 5% methanol, eluted with acetonitrile–ethanol and dissolved in formic acid–acetonitrile. The concentration of LAMZ in each sample was measured by liquid chromatography/mass spectrometry (LC/MS/MS, Shimadzu and Applied Biosystems).

### Micro-CT analysis

The fixed femur was radiologically scanned using a ScanXmate-A100S Scanner (Comscan Techno). A three-dimensional image of the femur was reconstructed, and the structural indices were calculated using TRI/3D-BON software (RATOC Systems).

### Histomorphometric analysis of the bone

For the analysis of osteoblastic bone formation and osteoclastic bone resorption in vivo, undecalcified tibiae were embedded in glycol methacrylate (GMA). The blocks of GMA were sectioned (5 μm) and subjected to toluidine blue staining or tartrate-resistant acid phosphatase (TRAP) staining. The parameters for bone formation and resorption in the secondary trabecular bone were assessed under microscopy (Axio Imager 2, Zeiss) and WinROOF 2013 v.1.4.1 software (Mitani). The width of the articular cartilage was measured using measurement software (BZ–X analyzer, Keyence).

### Histological analysis of the tissues

The fixed muscles were dehydrated and embedded in paraffin. Six-micrometer-thick sections were cut. The sections were stored at 4 °C until staining. After deparaffinization and hydration of the sections, the sections underwent staining. For hematoxylin and eosin staining, the sections were stained with hematoxylin (Muto Pure Chemicals) for 3 min followed by 2 min of staining with eosin (Wako). For modified Gomori’s trichrome staining, the sections were stained with hematoxylin (Muto Pure Chemicals) for 3 min followed by 10 min of staining with modified Gomori’s trichrome staining solution (Chromotrope 2 R, 9.6 mg·mL^−1^; Fast green FCF, 4.8 mg·mL^−1^; phosphotungstic acid hydrate 9.6 mg·mL^−1^; and acetic acid, 16 μL [pH 3.4]). The histological images were captured, and muscle fiber width and tendon width (the minor axes) were measured using measurement software (BZ–X analyzer, Keyence).^50^

### Isolation of cells from the mice

Osteoblast and osteoclast progenitors were harvested from neonatal and 6- to 8-week-old mice, respectively.^15,51^ Neonatal mice were sacrificed by anesthesia. After disinfection of mice using immersion in ethanol, the calvaria were dissected. Dissected tissue was digested in the following solution: 1 mg·mL^−1^ collagenase (Fujifilm Wako Pure Chemical) and 2 mg·mL^−1^ dispase (Fujifilm Wako Pure Chemical) in α-modified minimum essential medium (α-MEM) (Gibco). After digestion, tissue debris was removed, and osteoblastic cells were collected.

Bone marrow cells (BMCs) were harvested from the femur and tibia by introducing PBS into the bone marrow cavity. Erythrocytes were depleted using ammonium chloride (Sigma). Tissue debris was removed, and BMCs were collected. The collected cells underwent the experiments described below.

### Chemical compounds used in the in vitro experiments

The first screening was conducted using a chemical library (Stem Select^TM^ Small Molecule Regulators 384-Well Library I, Merck). Each compound was diluted to 10 mmol·L^−1^ so that the final concentration in the cell culture plate would become 10 μmol·L^−1^. The compounds used in the experiments other than primary screening were purchased individually: AICAR (Calbiochem), monastrol (Santa Cruz Biotechnology), PARP inhibitor XII (Calbiochem), AMI-5 (Calbiochem), 17b (Calbiochem), IWR-1-endo (Calbiochem), apocynin (Santa Cruz Biotechnology), L-165, 041 (Abcam Limited), linifanib (Cayman Chemical Company), SR18292 (Cayman Chemical Company), FK506 (Cayman Chemical Company) and KN93 (Cayman Chemical Company). These compounds were dissolved in DMSO (Sigma) so that their concentrations were 1 000 times greater than the final concentration.

### In vitro myotube differentiation

C2C12 cells were seeded onto cell culture plates at the following concentrations: 5.0 × 10^3^ cells per well for the 96-well plate and 1.5 × 10^4^ cells per well for the 48-well plate. After 1 day (Day 0), these cells were differentiated into myotubes by a differentiation medium, Dulbecco’s modified Eagle’s medium (DMEM) with 1 μg·mL^−1^ insulin and 2% horse serum. The differentiation medium was changed on Days 2, 3, 4, 5 and 6. Analyses were conducted on Day 7 unless otherwise indicated. LAMZ was added on Days 0, 2 and 4 at a concentration of 1 μmol·L^−1^. The PGC-1α inhibitor SR18292 was added on Days 0 and 2 at a concentration of 20 μmol·L^−1^. The calcineurin inhibitor FK506 was added on Days 0, 2, 4 and 6 at a concentration of 2 μmol·L^−1^.

Clonetics^TM^ Skeletal Muscle Myoblast Cell Systems (Lonza) was used for the analyses of human skeletal muscle. Cells with a population doubling number lower than seven were seeded onto cell culture plates at the following concentrations: 6.4 × 10^3^ cells per well for the 96-well plate and 1.5 × 10^4^ cells per well for the 48-well plate. After 1 day (Day 0), myotube differentiation was induced by adding DMEM-F12 with 2% horse serum (Lonza) and LAMZ (0.1 μmol·L^−1^). The differentiation medium was changed on Day 2. Analyses were conducted on Day 4.

For the primary screening, C2C12 cells were stimulated with the compounds included in the chemical library on Days 0, 2 and 4. On Day 7, these cells were fixed with 4% PFA at room temperature for 15 min. Cytosol was stained using HCS CellMask™ Deep Red Stain (Thermo Fisher Scientific), and nuclei were stained using Hoechst 33342 (Sigma). The number of cells and nuclei in each cell was measured with an IN Cell Analyzer 2000 (GE Healthcare). The number of cells and the number of multinuclear cells were normalized by cells stimulated with vehicle and are expressed as the relative cell number and relative myotube formation, respectively.

Myosin heavy or light chain expression was examined by immunocytofluorescence. Myotubes were fixed with 4% PFA at room temperature. The sections were then incubated with primary antibody solutions containing a mouse anti–myosin heavy chain antibody (MF20, dilution: 1/50, R&D Systems) or a mouse anti–myosin light chain antibody (MY20, dilution: 1/100, GeneTex) at room temperature for 3 h, followed by incubation with secondary antibody solutions containing a donkey anti–mouse IgG antibody conjugated with the fluorescence dye Alexa Fluor 594 (dilution: 1/500, Life Technologies) or Alexa Fluor 488 (dilution: 1/500, Life Technologies) for 1 hour. Nuclei were stained using Hoechst 33342 (Sigma). The stained images were obtained with a microscope (BZ–X analyzer, Keyence).

### In vitro osteoblast differentiation

Murine osteoblast progenitor cells (calvarial and MC-3T3-E1 cells) were seeded on cell culture plates at the following concentrations: 4.0 × 10^3^ cells per well for the 96-well plate and 7.5 × 10^3^ cells per well for the 48-well plate. After 1 day (Day 0), these cells underwent differentiation with an osteogenic medium containing 50 μg·mL^−1^ ascorbic acid, 10 nmol·L^−1^ dexamethasone and 10 mmol·L^−1^ β–glycerophosphate in α-MEM. The differentiation medium was changed every third day. LAMZ was added on Days 0, 3, 6, 9 and 12 at a concentration of 1 μmol·L^−1^. The PGC-1α inhibitor SR18292 was added on Days 0 and 3 at a concentration of 20 μmol·L^−1^. The CaMKII inhibitor KN93 was added on Days 0, 3, 6, 9 and 12 at a concentration of 2 μmol·L^−1^.

Poietics^TM^ human mesenchymal stem cells (Lonza) were used for analyses of human osteoblasts. These cells were seeded onto 48-well plates at a concentration of 7.2 × 10^3^ cells per well. After 1 day (Day 0), osteoblastogenesis was induced with hMSC differentiation basal medium-osteogenic (Lonza) supplemented with LAMZ (1 μmol·L^−1^). The differentiation medium was changed every third day.

For the secondary screening, MC3T3-E1 cells were stimulated with the candidate compounds for seven days. After fixation with 4% PFA at room temperature, these cells were supplemented with a substrate for alkaline phosphatase (ALP), *p*-nitrophenylphosphate disodium (Fujifilm Wako Pure Chemical), and incubated for 30 min at 37 °C without detaching the cells from the plate, unlike the conventional procedure.^14,15^ The catalysis of the substrate was halted by the stop solution 0.2 mmol·L^−1^ sodium hydroxide. A plate reader (Bio-Rad Laboratories) was used for the detection of the *p*-nitrophenylphosphate disodium catabolite *p*-nitrophenol (wavelength: 405 nm).

ALP staining and quantification were performed as follows. Osteoblasts were fixed with 4% PFA for 15 min on ice. After the cells were rinsed with PBS, they were stained for 15 min with ALP staining solution (Napthol AS-MX phosphate, 0.06 mg·mL^−1^; *N*,*N*-dimethylformamide, 1%; and Fast blue BB salt, 1 mg·mL^−1^ in 0.1 mmol·L^−1^ Tris-HCl [pH 8.0]). The staining solution was washed away with dH_2_O. The stained cells were air-dried. The stained images were obtained under microscopy (BZ–X analyzer, Keyence). The images were then converted into black and white images for the quantification of the intensity (Photoshop 2020, Adobe).

Mineralization was detected by Alizarin Red S (ARS) staining on Day 14. Cells were fixed with 4% PFA for 15 min on ice. After the cells were rinsed with dH_2_O, ARS staining was performed (0.02 g·mL^−1^ Alizarin Red S in dH_2_O [pH 4.2]). The staining solution was washed away with dH_2_O and air dried. The stained images were obtained under microscopy (BZ–X analyzer, Keyence).

### In vitro osteocyte differentiation

The murine immature osteocyte cell line IDG-SW3/1G9 was used in the experiments.^52^ These cells were seeded on a collagen-coated 24-well plate (Corning) at a concentration of 2.5 × 10^4^ cells per well. After 1 day (Day 0), these cells underwent differentiation with differentiation medium containing 50 μg·mL^−1^ ascorbic acid and 4 mmol·L^−1^ β–glycerophosphate in α-MEM. The differentiation medium was changed every third day. LAMZ was added on Days 0 and 9 at a concentration of 0.1 μmol·L^−1^.

### Analyses of mitochondria in cultured cells

C2C12 cells and MC3T3-E1 cells treated with LAMZ (1 μmol·L^−1^) were analyzed for their mitochondrial content on Days 3 and 6. MitoTracker^®^ Deep Red (Thermo Fisher Scientific) was incorporated into these cells for 30 min before fixation. Fixation was conducted with 4% PFA at room temperature for 15 min. Nuclei were stained using Hoechst 33342 (Sigma). The stained images were obtained under microscopy (BZ–X analyzer, Keyence).

### Visualization of intracellular calcium in vitro

C2C12 cells or MC3T3-E1 cells were seeded in a 96-well plate at a concentration of 5.0 × 10^3^ cells per mL. One day after incubation, these cells were loaded with Fluo 4 AM (Dojindo Laboratories) for 1 h. The loaded cells were then stimulated with LAMZ (10 μmol·L^−1^) for 4 h. Visualized intracellular calcium was acquired and quantified under microscopy with measurement software (BZ–X analyzer, Keyence).

### Gene knockdown

The plasmids for producing lentivirus that code shRNAs targeting *Mef2c* and *gfp* (control) were purchased from Sigma-Aldrich. These plasmids were transfected into HEK293T cells using FuGENE HD transfection reagent (Promega). The number of lentiviral particles (LP) was calculated by measuring p24 expression with an enzyme–linked immunoassay (ELISA) kit (TaKaRa). The viral titer was calculated by assuming 1 inclusion forming unit (IFU) = 100 LP. The lentiviruses were used to infect C2C12 cells or MC3T3-E1 cells at a multiplicity of infection (MOI) of 100. One day after infection, myogenic or osteogenic differentiation was induced with or without LAMZ as described above. Puromycin (2 μg·mL^−1^) was added at the same time as LAMZ to eliminate uninfected cells.

### In vitro osteoclast differentiation from mouse BMCs

Primary BMCs were seeded on cell culture plates at the following concentrations: 2.5 × 10^5^ cells per well for the 96-well plate, 1.3 × 10^5^ cells per well for the 48-well plate and 2.0 × 10^5^ cells per well for the 24-well plate. BMCs were expanded in α-MEM with 10 ng·mL^−1^ macrophage colony–stimulating factor (M-CSF) (R&D systems) for 2 days before the induction of differentiation. These cells were stimulated with differentiation medium containing 10 ng·mL^−1^ M-CSF and 25 ng·mL^−1^ RANKL (PeproTech) in α-MEM at Day 0. The differentiation medium was changed on Day 2. Cells were analyzed on Day 3 unless otherwise indicated.

Tertiary screening was conducted using BMCs harvested from CAG–CAT–*lacZ*^Tg/Wt^*Ctsk*^*Cre*/Wt^ mice. On Day 3, the differentiation medium was washed away using PBS, and a substrate of β-galactosidase, 6-O-β-galactopyranosyl-luciferin (Promega), was added. After 30 min of incubation, catabolites were detected based on chemiluminescence (wavelength: 560 nm) using a plate reader (PerkinElmer).

TRAP^+^ multinuclear cells were detected by TRAP staining. On Day 3, osteoclasts were fixed with 4% PFA for 15 min on ice. Membrane delipidation was performed using an acetone–ethanol solution (1:1 v/v) for 30 s. TRAP staining solution was then applied, and the cells were incubated for approximately 5 min at room temperature until the cells positive for TRAP became pale pink. The composition of the staining solution was as follows: 0.1 mg·mL^−1^ naphthol AS-MX phosphate (Sigma-Aldrich), 10 μL·mL^−1^ *N*,*N*-dimethylformamide (Nacalai Tesque), and 0.6 mg·mL^−1^ fast red violet LB salt (Sigma-Aldrich) in a TRAP buffer (5.44 g·L^−1^ sodium acetate and 10.5 g·L^−1^ sodium tartrate). The stained images were obtained under a microscope (BZ–X analyzer, Keyence), and TRAP^+^ cells with 10 or more nuclei were regarded as osteoclasts.

Apoptotic cells were detected by TdT-mediated dUTP nick-end labeling (TUNEL) assay. BMCs that had undergone osteoclastogenesis in the presence of LAMZ (0.01 μmol·L^−1^) were fixed on Day 2. The fragmented DNA of the apoptotic cells was detected using the DeadEnd^TM^ Fluorometric TUNEL System (Promega) according to the manufacturer’s instructions. Fluorescein-12-dUTP was incorporated into the fragmented DNA at the 3’ end, which was detected under microscopy (BZ–X analyzer, Keyence).

Cleaved caspases 3 and 8 were detected by immunocytofluorescence. BMCs were fixed with 4% PFA at 4 °C on Day 2. The cells were then incubated with a primary antibody solution containing a rabbit anti–cleaved caspase 3 antibody (polyclonal, dilution: 1/400, Cell Signaling Technology) or a rabbit anti–cleaved caspase 8 antibody (D5B2, dilution: 1/400, Cell Signaling Technology). Nuclei were stained using Hoechst 33342 (Sigma). The stained images were obtained under microscopy (BZ–X analyzer, Keyence).

### Quantitative reverse transcriptase–polymerase chain reaction (qRT-PCR)

The total RNA of mouse tissues was extracted as described above, and that of the cultured cells was extracted using a Maxwell RSC simple RNA Tissue Kit (Promega). cDNA was synthesized from the extracted RNA using ReverTra Ace^®^ (TOYOBO). qRT-PCR analysis was performed with SYBR Green Real-time PCR Master Mix (TOYOBO) using a Light Cycler apparatus (Bio-Rad Laboratories). Gene expression was calculated using the ΔΔCt method, and *Gapdh* expression was used for normalization. The primer sequences are listed below: *Gapdh*, 5′–ACCCAGAAGACTGTGGATGG–3′ and 5′–CACATTGGGGGTAGGAACAC–3′; *Myod1*, 5′–AACCCCAATGCGATTTATCA–3′ and 5′–CGAAAGGACAGTTGGGAAGA–3′; *Myog*, 5′–CTGCACTCCCTTACGTCCAT–3′ and 5′–ACCCAGCCTGACAGACAATC–3′; *Klf5*, 5′–GGTTGCACAAAAGTTTATAC–3′ and 5′–GGCTTGGCGCCCGTGTGCTTCC–3′; *Mef2c*, 5′–CGCAGGGAATGGATACGGCAAC–3′ and 5′–GGGATAAGAACGCGGAGATCTGG–3′; *Runx2*, 5′–CCCAGCCACCTTTACCTACA–3′ and 5′–TATGGAGTGCTGCTGGTCTG–3′; *Sp7*, 5′–ACTGGCTAGGTGGTGGTCAG–3′ and 5′–GGTAGGGAGCTGGGTTAAGG–3′; *Alpl*, 5′–AACCCAGACACAAGCATTCC–3′ and 5′–GCCTTTGAGGTTTTTGGTCA–3′; *Sost*, 5′–GGAATGATGCCACAGAGGTCA–3′ and 5′–CGTCATAGGGATGGTGGGGA–3′; *Ppargc1a*, 5′–AGTCCCATACACAACCGCAG–3′ and 5′–ACCCTTGGGGTCATTTGGTG–3′; *Fos*, 5′–CAGCCTTTCCTACTACCATTCC-3′ and 5′–ACAGATCTGCGCAAAAGTCC–3′; *Mef2c*, 5′–CGCAGGGAATGGATACGGCAAC-3′ and 5′–GGGATAAGAACGCGGAGATCTGG-3′; *Mef2c* (for the analyses of knockdown efficiency), 5′–TATGTGCCGTGTGTGGAAAA-3′ and 5′–AGTGCTAAGCGTATCTCAGC-3′; *GAPDH*, 5′–TGACCACAGTCCATGCCATC–3′ and 5′–GATGATGTTCTGGAGAGCCCC–3′; *MYOD1*, 5′–GCCACAACGGACGACTTCTA–3′ and 5′–CGAGTGCTCTTCGGGTTTCA–3′; *MYOG*, 5′–ATCATCTGCTCACGGCTGAC–3′ and 5′–GGGCATGGTTTCATCTGGGA–3′; *PPARGC1A*, 5′–ACACTTTGCGCAGGTCAAACG–3′ and 5′–TGGTGGAAGCAGGGTCAAAG–3′; *RUNX2*, 5′–ACTGGGCCCTTTTTCAGACC–3′ and 5′–GGACATACCGAGGGACATGC–3′; *SP7*, 5′–ATCCAGCCCCCTTTACAAGC–3′ and 5′–TGAGTGGGAAAAGGGAGGGTA–3′.

### RNA sequencing

The total RNA of cultured calvarial cells was extracted using a Maxwell 16 LEV simplyRNA Tissue Kit (Promega). Data were acquired on an Ion Proton (Thermo Fisher) and analyzed using CLC Genomics Workbench (CLC). *k*-means clustering and Gene Ontology enrichment analysis were performed using an online tool, iDEP.91.^53^

### Western blotting analysis

For analysis of PLCγ1 phosphorylation, C2C12 cells or MC3T3-E1 cells were stimulated with LAMZ (10 μmol·L^−1^) after serum starvation (6 hours). The total proteins of these cells were extracted with lysis buffer containing Triton X–100 (1%), NaCl (150 mmol·L^−1^), Tris-HCl (50 mmol·L^−1^), EDTA (1 mmol·L^−1^), sodium deoxycholate (0.5%), Na_4_P_2_O_7_⋅10H_2_O (40 mmol·L^−1^), NaF (50 mmol·L^−1^), Na_3_VO_4_ (1 mmol·L^−1^), PMSF (2 mmol·L^−1^) and cOmplete^TM^ Protease Inhibitor Cocktail (Roche Bioscience). The extracted proteins underwent SDS–PAGE and were transferred to PVDF membranes (pore size: 0.45 μm, Merck), followed by immunoblotting using the following antibodies: PLCγ1 (1/1 000, CST), phospho–PLCγ1 S1248 (1/1 000, CST), and β-actin (1/5 000, Merck). The blots were visualized using the following reagents: horseradish peroxidase (HRP)–linked anti–mouse IgG, HRP–linked anti–rabbit IgG (1/5 000, GE Healthcare), and a luminol reagent (Nacalai Tesque). Densitometric analysis of the bands was conducted using Photoshop 2020 (Adobe).

### Statistical Analysis

All of the data are representative of more than three independent experiments and were initially tested with the F test or Bartlette’s test for normality distribution. If homoscedasticity could be assumed, they were analyzed with a parametric test using Student’s *t* test, one-way analysis of variance (ANOVA) followed by Dunnett’s or Tukey’s multiple-comparison test or two–way ANOVA followed by Tukey’s multiple-comparison test. In cases in which homoscedasticity could not be assumed, Welch’s *t* test or Brown–Forsythe ANOVA test followed by Dunnett’s T3 test were applied. Differences with a *p* value of <0.05 were considered significant (*P < 0.05; **P < 0.01; ***P < 0.001; ****P < 0.000 1; N. S., not significant, throughout the paper). All data are presented as the mean ± standard error of the mean values. All statistical analyses were performed with Prism 8 (GraphPad Software).

## Supplementary information

Supplementary figure 1

Supplementary figure 2

Supplementary figure 3

Supplementary figure 4

Supplementary figure 5

Supplementary figure 6

Supplementary figure 7

Supplementary figure 8

Supplementary figure 9

## Data Availability

The data that support the findings of this study are openly available in GEO at https://www.ncbi.nlm.nih.gov/geo/, reference number GSE188343.
