# Supplementary material for: Simultaneous augmentation of muscle and bone by locomomimetism through calcium-PGC-1α signaling
Source: Bone Res. 2022 Aug 3;10:52. doi: 10.1038/s41413-022-00225-w (PMC9345981; doi:10.1038/s41413-022-00225-w)
Supplement: Supplementary file 1 — Supplementary figure 1 [file 41413_2022_225_MOESM1_ESM.pdf]

Supplementary Fig. 1

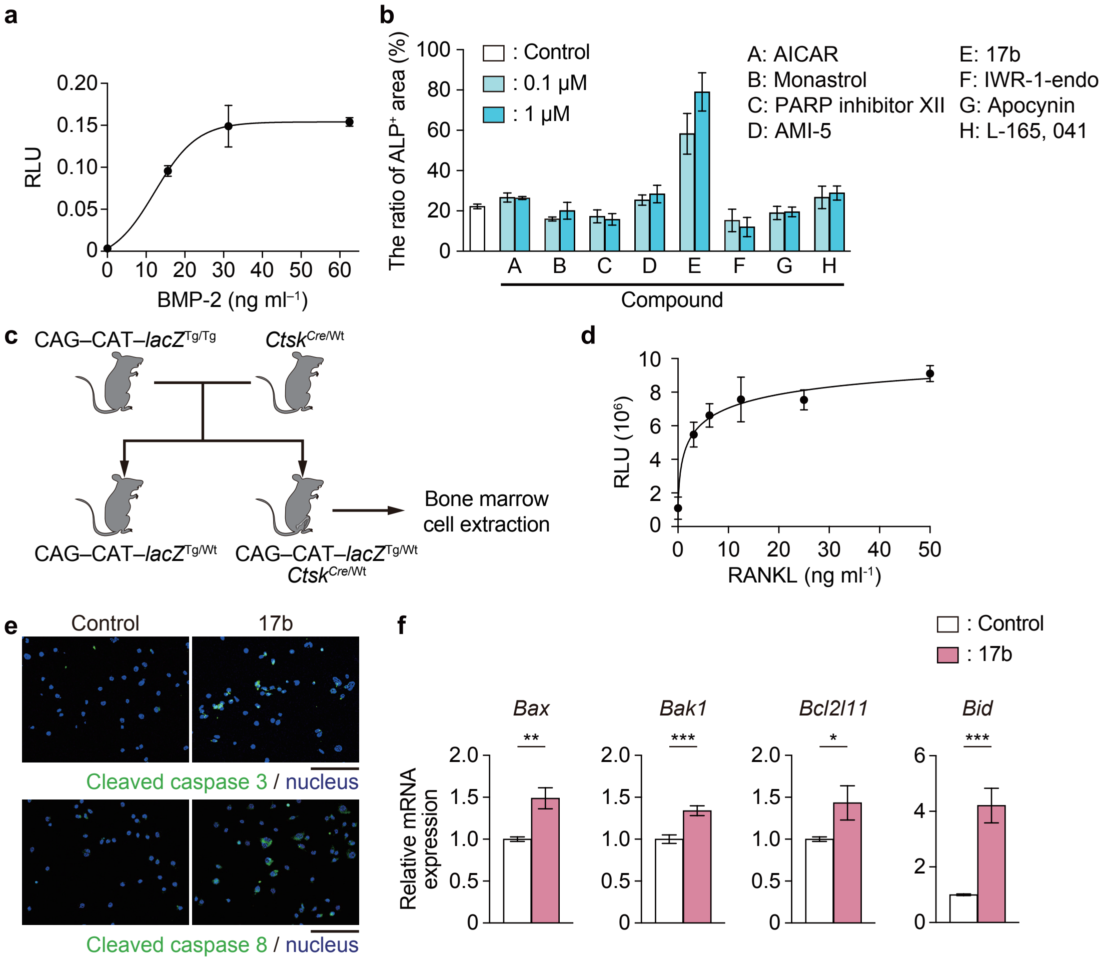

Supplementary Fig. 1 Establishment of the screening systems used to identify a novel drug for reinforcing both muscle and bone. (a) A novel quantification method of osteoblastogenesis. The activity of alkaline phosphatase (ALP) was quantified according to the degradation rate of its substrate (see methods). **(b)** Quantification of ALP activity based on colorimetric analysis. **(c)** Generation of the mice used for the novel quantification method of osteoclastogenesis. Mice expressing β-galactosidase in osteoclasts by the *Ctsk* promoter (CAG–CAT–*lacZ*$^{Tg/Wt}$*Ctsk*$^{Cre/Wt}$ mice) were generated by crossing CAG–CAT–*lacZ*$^{Tg/Tg}$ female mice and *Ctsk*$^{Cre/Wt}$ male mice. **(d)** Osteoclastogenesis of bone marrow cells of CAG–CAT–*lacZ*$^{Tg/Wt}$*Ctsk*$^{Cre/Wt}$ mice was quantified by *Ctsk* expression, which is indicated by β-galactosidase activity. **(e)** Immunocytofluorescence showing the expression of the cleaved caspase 3 and cleaved caspase 8 in bone marrow cells treated with 17b. The cleaved caspases (green); and nuclei (blue). Scale bar, 100 μm. **(f)** mRNA expression of proapoptotic genes in osteoclast progenitors treated with LAMZ. The data of the effects of 17b were obtained from 3 independent experiments with replicates of 2 or 3 wells. Statistical analyses were carried out using Student's *t* test or Welch's *t* test. The error bars show the mean ± s.e.m. *$p < 0.05$; **$p < 0.01$; ***$p < 0.001$.
