# Supplementary material for: Simultaneous augmentation of muscle and bone by locomomimetism through calcium-PGC-1α signaling
Source: Bone Res. 2022 Aug 3;10:52. doi: 10.1038/s41413-022-00225-w (PMC9345981; doi:10.1038/s41413-022-00225-w)
Supplement: Supplementary file 2 — Supplementary figure 2 [file 41413_2022_225_MOESM2_ESM.pdf]

Supplementary Fig. 2

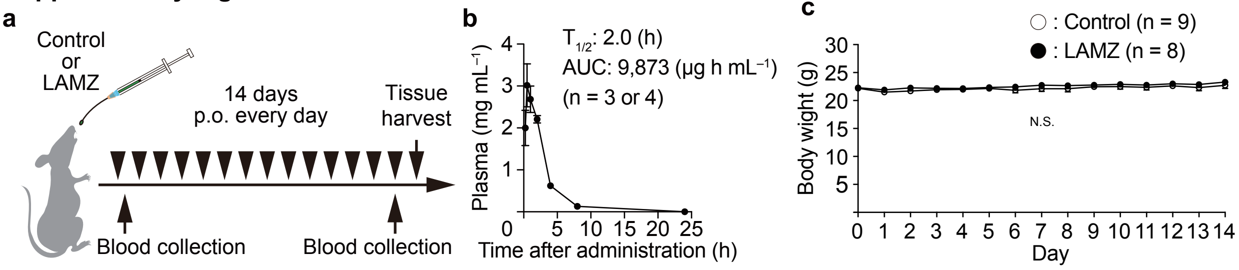

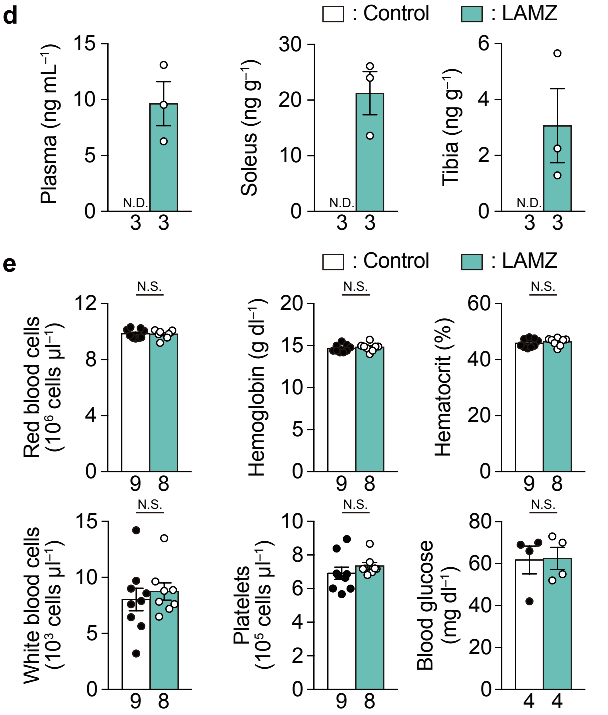

f

	Control	LAMZ	*t* test
Na (mEq/L)	151±0.7	149±1.0	N.S.
K (mEq/L)	4.6±0.35	5.1±0.40	N.S.
Cl (mEq/L)	111±0.3	109±0.9	N.S.
Ca (mg/dL)	9.4±0.11	9.5±0.12	N.S.
IP (mg/dL)	7.9±0.45	8.8±0.47	N.S.
TP (g/dL)	4.6±0.13	4.6±0.09	N.S.
ALB (g/dL)	3.0±0.07	3.2±0.08	N.S.
AST (IU/L)	274±59.1	307±65.7	N.S.
ALT (IU/L)	54±15.0	95±26.4	N.S.
LDH (IU/L)	718±181	633±113	N.S.
T-CHO (mg/dL)	101±8.1	98±5.2	N.S.
TG (mg/dL)	122±13.4	101±7.7	N.S.
HDL-C (mg/dL)	50±2.7	54±2.4	N.S.
BUN (mg/dL)	30.8±1.78	29.1±1.34	N.S.
CRE (mg/dL)	0.11±0.005	0.11±0.008	N.S.

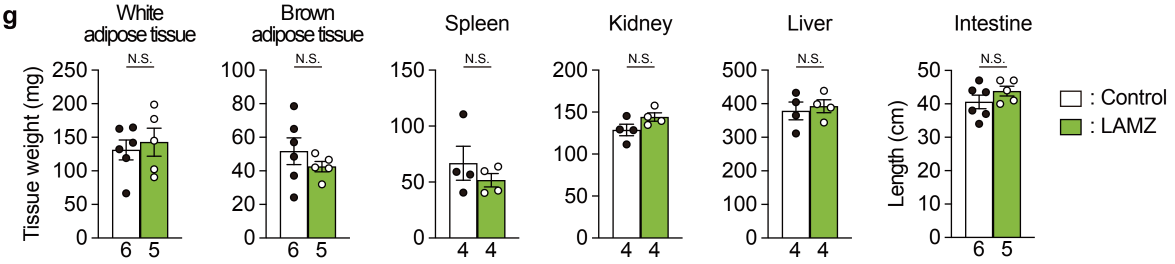

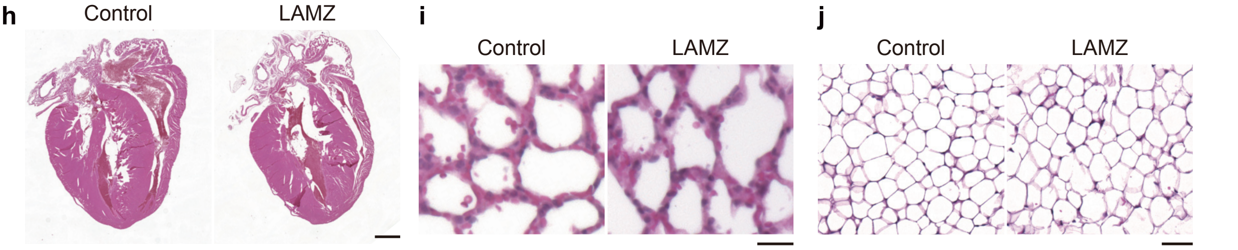

Supplementary Fig. 2 General effects of LAMZ after 14 days of oral administration. (a) Schematic diagram of the experiment. **(b)** Plasma concentration of LAMZ after oral administration. Peripheral blood was collected 0.25, 0.5, 1, 2, 4, 8 and 24 h after the administration. **(c)** Body weight of mice treated with LAMZ or control emulsion. **(d)** Concentration of LAMZ in the plasma, soleus muscle and tibia after sacrifice. N.D., not detected. **(e)** Blood cell count and blood glucose before sacrifice. **(f)** Serum biochemistry profile of the mice treated orally with LAMZ or control emulsion. **(g)** The tissue weight of the fat, spleen, kidney and liver as well as the length of the intestine in mice treated with LAMZ. **(h)** Representative histological images of the heart. **(i)** Representative histological images of the lung. **(j)** Representative histological images of white adipose tissue. Sections were stained with hematoxylin and eosin. Scale bars, 1 mm in **(h)**, 50 μm in **(i)** and 20 μm in **(j)**. 4 sections per mouse and 4 mice in each group were analyzed. The number of biological replicates is described below each bar. For the multiple comparisons of the body weight, two-way ANOVA and Tukey's multiple–comparison test were applied. For the comparison of 2 groups, statistical analyses were carried out using Student's *t* test. The error bars show the mean ± s.e.m. N.S., not significant.
