# Supplementary material for: Simultaneous augmentation of muscle and bone by locomomimetism through calcium-PGC-1α signaling
Source: Bone Res. 2022 Aug 3;10:52. doi: 10.1038/s41413-022-00225-w (PMC9345981; doi:10.1038/s41413-022-00225-w)
Supplement: Supplementary file 3 — Supplementary figure 3 [file 41413_2022_225_MOESM3_ESM.pdf]

Supplementary Fig. 3

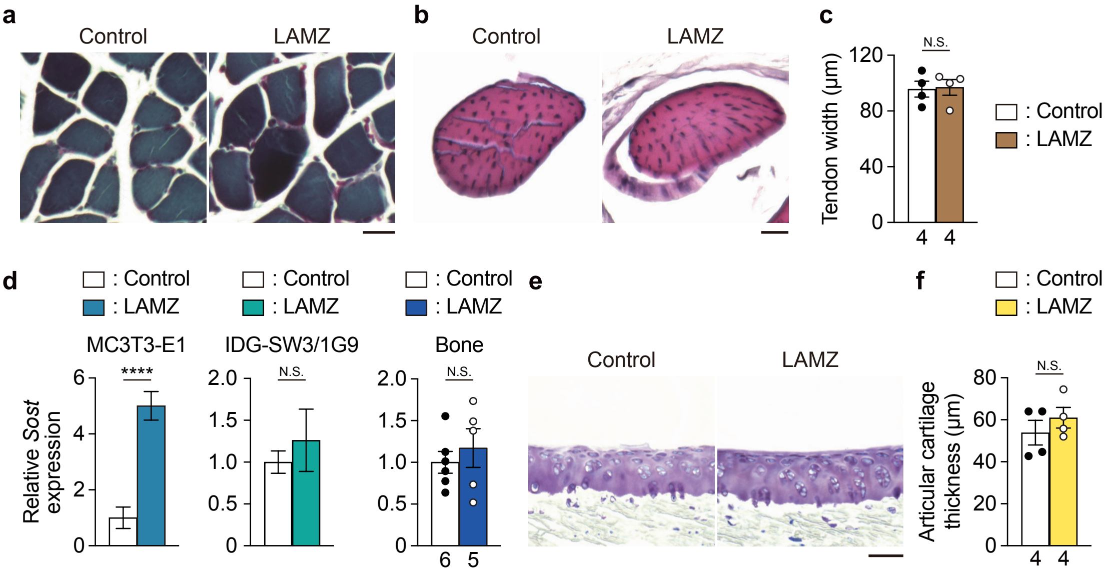

Supplementary Fig. 3 Effects of LAMZ on musculoskeletal tissues after 14 days of oral administration. (a) Representative histological images of the soleus muscle of mice orally treated with LAMZ or a control emulsion. Cross sections were stained by Modified Gomori's Trichrome staining method. **(b)** Representative histological images of the carpal tendon. Cross sections were stained using hematoxylin and eosin. **(c)** Mean value of the width of the tendon. **(d)** mRNA expression of *Sost* in the bone cells and bone tissue. **(e)** Representative histological images of the tibial articular cartilage. Coronal sections were stained using toluidine blue. **(f)** Thickness of the articular cartilage. Scale bars, 20 µm in **(a)** and **(b)**; and 50 µm in **(e)**. 4 sections per mouse and 4 mice in each group were analyzed. The number of biological replicates is described below each bar. Statistical analyses were carried out using Student's t test or Welch's t test. The error bars show the mean ± s.e.m. $*p < 0.05$; $****p < 0.0001$; N.S., not significant.
