# Supplementary material for: Simultaneous augmentation of muscle and bone by locomomimetism through calcium-PGC-1α signaling
Source: Bone Res. 2022 Aug 3;10:52. doi: 10.1038/s41413-022-00225-w (PMC9345981; doi:10.1038/s41413-022-00225-w)
Supplement: Supplementary file 4 — Supplementary figure 4 [file 41413_2022_225_MOESM4_ESM.pdf]

Supplementary Fig. 4

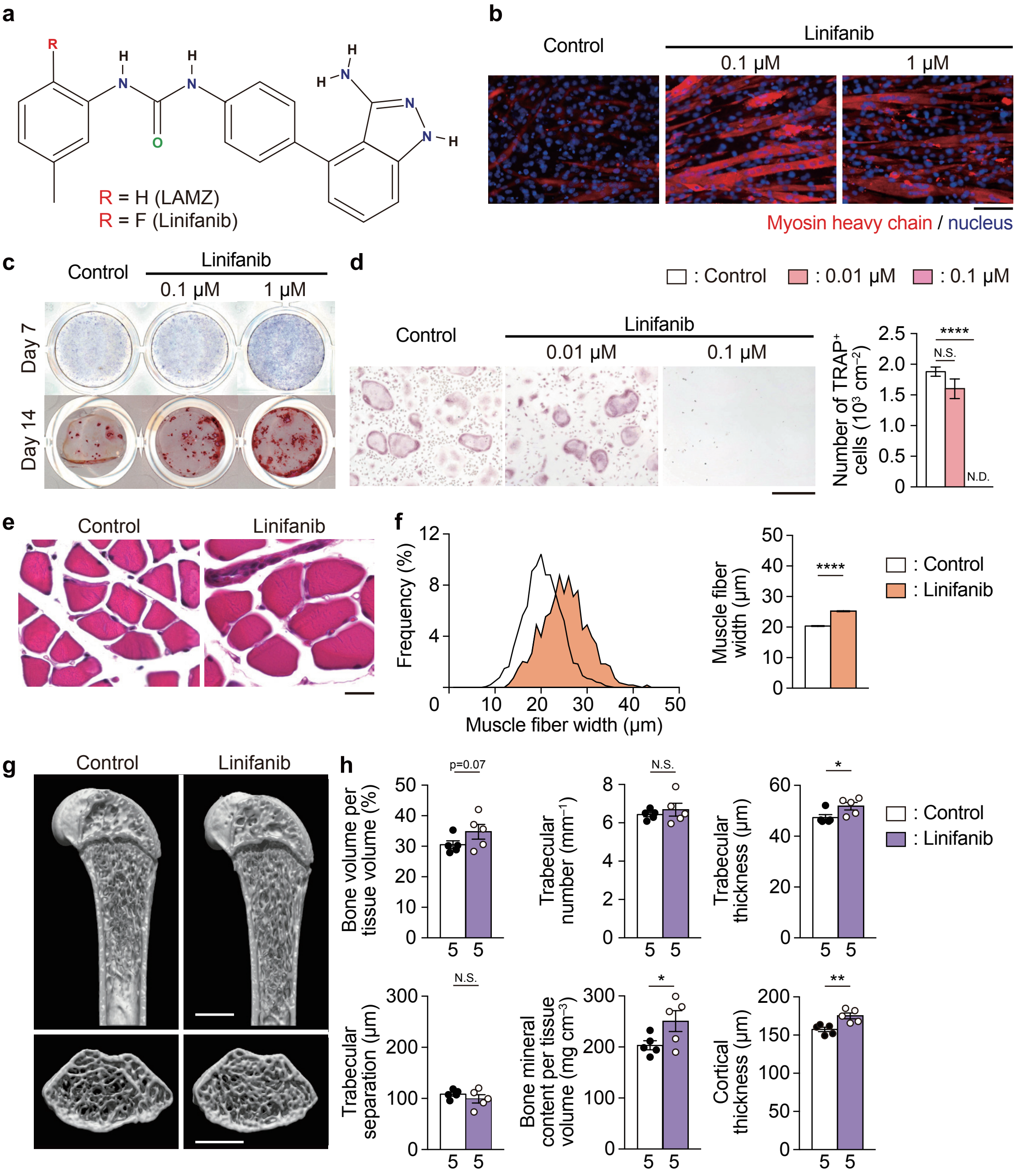

Supplementary Fig. 4 A LAMZ analogue linifanib similarly reinforces both muscle and bone. (a) Structural formula of LAMZ and linifanib. **(b)** Representative immunocytofluorescence images of C2C12 cells stimulated with linifanib. Myosin heavy chain (red); and nuclei (blue). Scale bar, 100 μm. **(c)** Representative images of alkaline phosphatase (ALP) staining (upper) and mineralization (lower) of calvarial cells stimulated with linifanib during osteoblastic differentiation. **(d)** Representative images of tartrate–resistant acid phosphatase (TRAP) staining of bone marrow cells (BMCs) stimulated with linifanib. Scale bar, 100 μm. The bar graph shows the number of TRAP$^+$ multinucleated cells. N.D., not detected. **(e)** Representative histological images of the soleus muscle of mice orally administered linifanib or a control emulsion. Cross sections of the muscle were stained with hematoxylin and eosin. Scale bar, 20 μm. **(f)** Distribution and mean value of the width of the soleus muscle fibers. 4 sections per mouse and 4 mice in each group were analyzed. In total, the numbers of fibers measured were 1,419 and 1,084, respectively. **(g)** Representative micro-computed tomography (CT) images of the femur of mice orally treated with linifanib or the control emulsion. Upper, sagittal section; and lower, transverse section of the metaphyseal area. Scale bar, 1 mm. **(h)** Bone parameters obtained by micro-CT analyses. The data of in vitro experiments were obtained from 3 independent experiments with replicates of 2 or 3 wells. The number of biological replicates is described below each bar. For the multiple comparisons, one-way ANOVA and Dunnett's multiple–comparison test or Brown–Forsythe ANOVA test and Dunnett's T3 test were applied. For the comparison of 2 groups, statistical analyses were carried out using Student's *t* test. Error bars show the mean ± s.e.m. *$p < 0.05$; **$p < 0.01$; ****$p < 0.0001$; N.S., not significant.
