# Supplementary material for: Simultaneous augmentation of muscle and bone by locomomimetism through calcium-PGC-1α signaling
Source: Bone Res. 2022 Aug 3;10:52. doi: 10.1038/s41413-022-00225-w (PMC9345981; doi:10.1038/s41413-022-00225-w)
Supplement: Supplementary file 5 — Supplementary figure 5 [file 41413_2022_225_MOESM5_ESM.pdf]

Supplementary Fig. 5

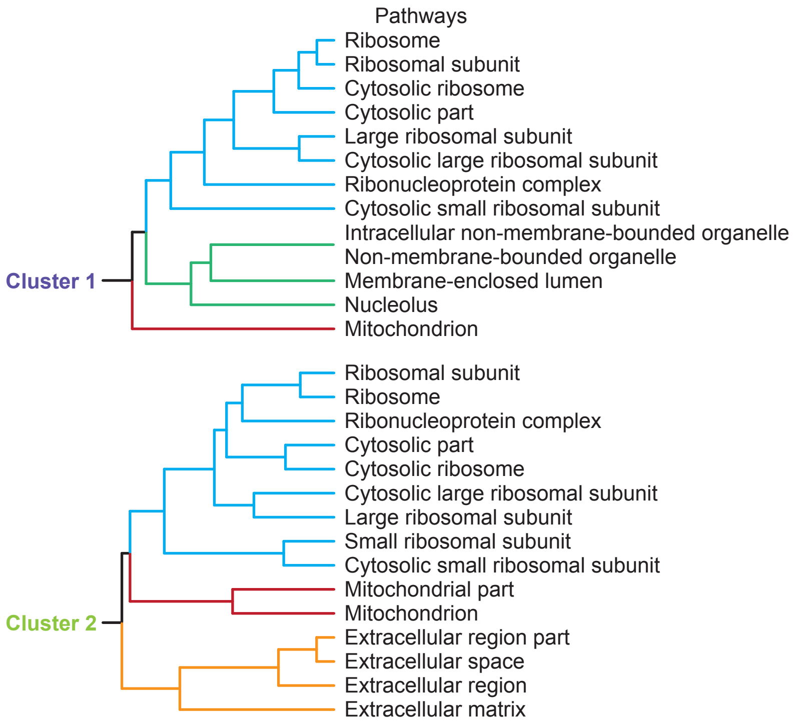

Supplementary Fig. 5 LAMZ treatment upregulates mitochondrial genes. Gene ontology analysis was based on a comprehensive gene expression analysis procedure. The enriched pathways in the clusters 1 and 2 (see **Fig. 3a**) consist of genes highly expressed in LAMZ–treated cells. The Gene Ontology (GO) terms (Cellular component) annotated to the genes were analyzed and the tree diagrams of the GO term hierarchy was constructed using the online tool iDEP.91.
