# Supplementary material for: Simultaneous augmentation of muscle and bone by locomomimetism through calcium-PGC-1α signaling
Source: Bone Res. 2022 Aug 3;10:52. doi: 10.1038/s41413-022-00225-w (PMC9345981; doi:10.1038/s41413-022-00225-w)
Supplement: Supplementary file 6 — Supplementary figure 6 [file 41413_2022_225_MOESM6_ESM.pdf]

Supplementary Fig. 6

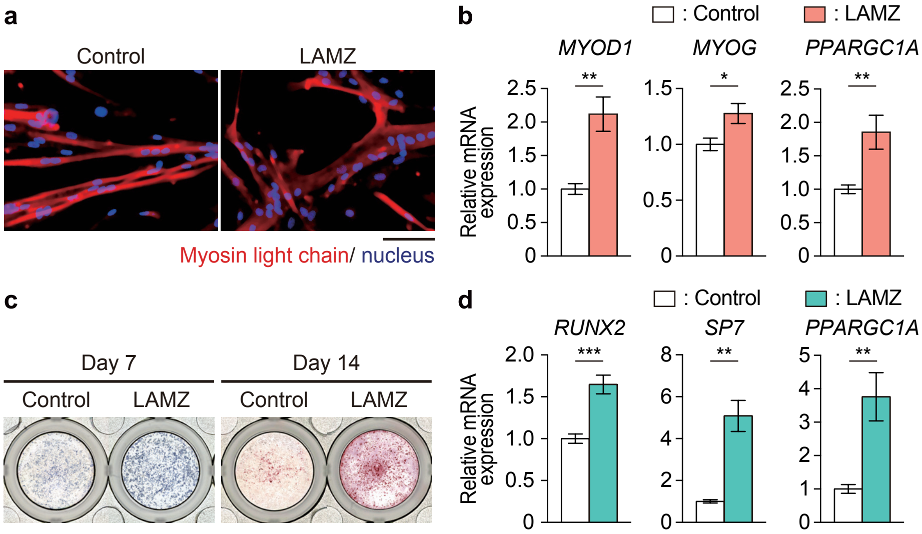

Supplementary Fig. 6 Effects of LAMZ on human myocytes and osteoblasts. **(a)** Representative immunocytofluorescence images of human myocytes stimulated with LAMZ. Myosin light chain (red); and nuclei (blue). **(b)** mRNA expression of myogenic genes and *Ppargc1a*. **(c)** Representative images of alkaline phosphatase (ALP) staining and Alizarin Red S staining of human mesenchymal stem cells stimulated with LAMZ. **(d)** mRNA expression of osteoblastic genes and *Ppargc1a*. Experiments were repeated 3 times with replicates of 2 or 3 wells. Scale bar, 100 μ m. Statistical analyses were carried out using Student's *t* test or Welch's *t* test. The error bars show the mean ± s.e.m. *$p < 0.05$; **$p < 0.01$; ***$p < 0.001$.
