# Supplementary material for: Simultaneous augmentation of muscle and bone by locomomimetism through calcium-PGC-1α signaling
Source: Bone Res. 2022 Aug 3;10:52. doi: 10.1038/s41413-022-00225-w (PMC9345981; doi:10.1038/s41413-022-00225-w)
Supplement: Supplementary file 7 — Supplementary figure 7 [file 41413_2022_225_MOESM7_ESM.pdf]

Supplementary Fig. 7

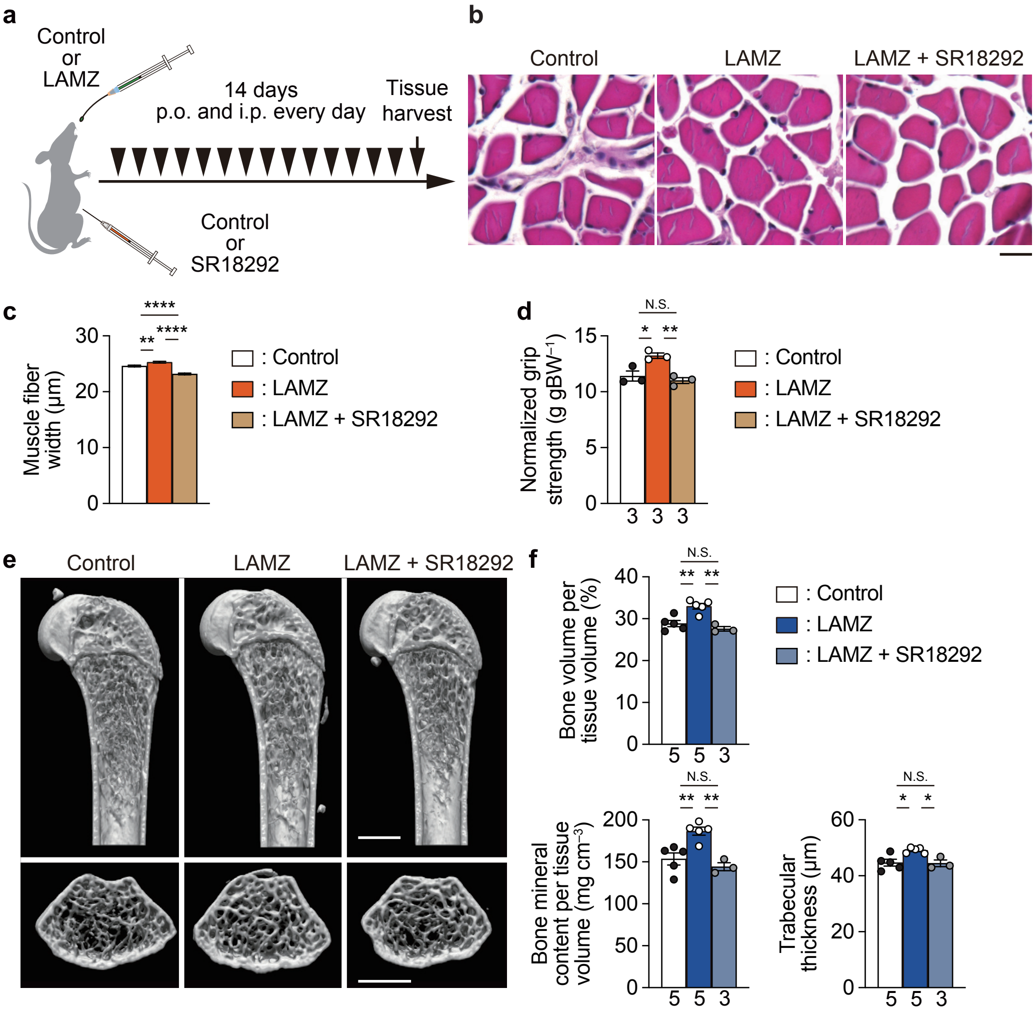

Supplementary Fig. 7 Inhibition of PGC-1α negates the anabolic effects of LAMZ on muscle and bone *in vivo*. **(a)** Schematic diagram of the experiment. **(b)** Representative histological images of the soleus muscle of mice subcutaneously injected with SR18292 or control solution, in addition to receiving oral administration of LAMZ or a control emulsion. Cross sections of the muscle were stained with hematoxylin and eosin. Scale bar, 20 µm. **(c)** Distribution and mean width of the soleus muscle fibers. 3 mice in each group were analyzed. In total, the numbers of fibers measured were 940, 958 and 990, respectively. **(d)** Grip strength of the mice. **(e)** Representative micro-computed tomography (CT) images of the femur of mice subcutaneously injected with SR18292 or control solution, in addition to receiving oral administration of LAMZ or a control emulsion. Upper, sagittal section; and lower, transverse section of the metaphyseal area. Scale bar, 1 mm. **(f)** Bone parameters obtained by micro-CT analyses. Statistical analyses were carried out using one-way ANOVA and Tukey's multiple–comparison tests. The number of biological replicates is described below each bar. Error bars show the mean ± s.e.m. $*p < 0.05$; $**p < 0.01$; $****p < 0.0001$; N.S., not significant.
