# Supplementary material for: Simultaneous augmentation of muscle and bone by locomomimetism through calcium-PGC-1α signaling
Source: Bone Res. 2022 Aug 3;10:52. doi: 10.1038/s41413-022-00225-w (PMC9345981; doi:10.1038/s41413-022-00225-w)
Supplement: Supplementary file 8 — Supplementary figure 8 [file 41413_2022_225_MOESM8_ESM.pdf]

Supplementary Fig. 8

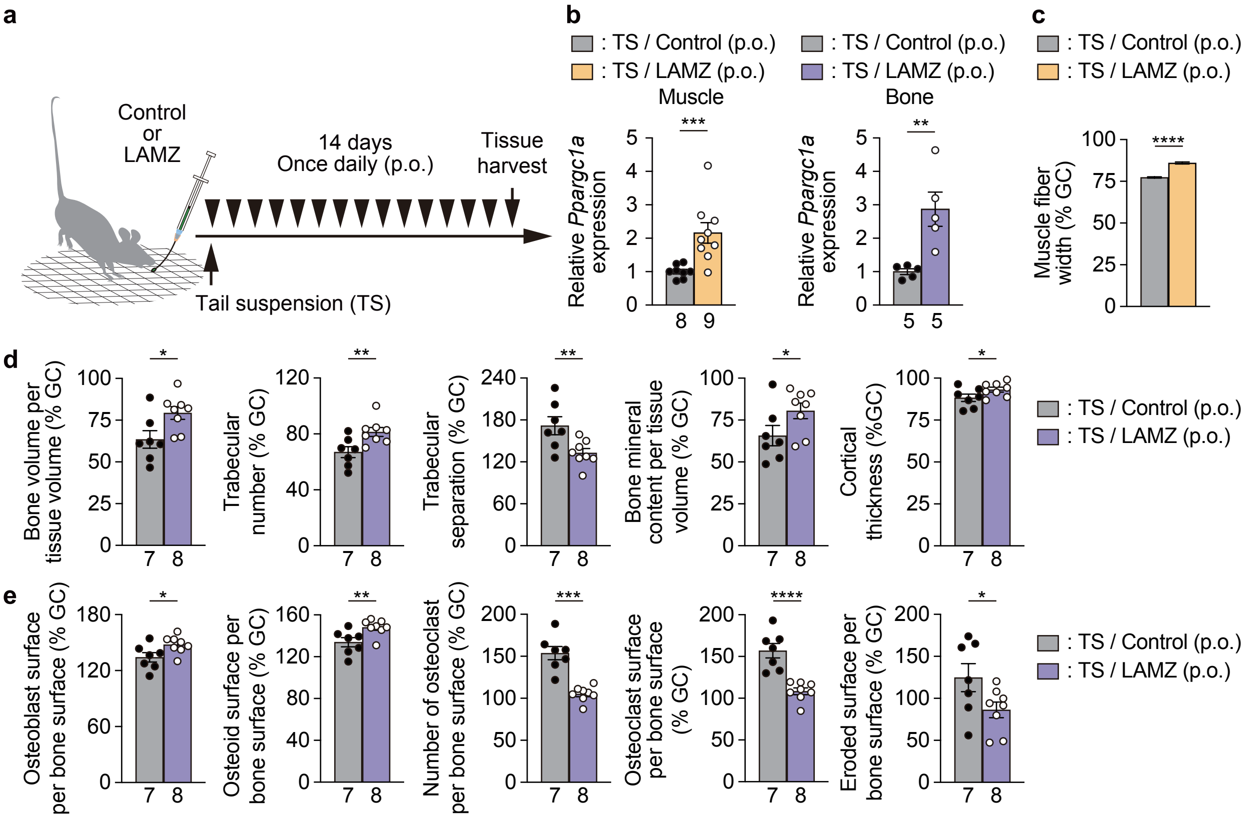

Supplementary Fig. 8 LAMZ improves both the muscle and bone of mice with locomotor frailty. (a) Schematic diagram of the experiment in **Fig. 6**. **(b)** mRNA expression of *Ppargc1a* in the muscle and the bone of tail suspension (TS) mice orally treated with LAMZ or the control emulsion. **(c)** The ratio of the fiber width in the soleus muscle of the mice that underwent an unloading model and were orally treated with LAMZ or a control emulsion, compared to that of ground control mice treated with a control emulsion. **(d)** The ratio of bone parameters obtained by micro-computed tomography (CT) analyses compared to that of ground control mice treated with a control emulsion. **(e)** The ratio of parameters of osteoblastic bone formation and osteoclastic bone resorption obtained by bone morphometric analysis compared to that of ground control mice treated with a control emulsion. The parameters were calculated using mice in experiment groups in which ground control (GC) and TS were conducted at the same time. Statistical analyses were carried out using Student's *t* test or Welch's *t* test. The number of biological replicates is described below each bar. The error bars show the mean ± s.e.m. $*p < 0.05$; $**p < 0.01$; $***p < 0.01$; $****p < 0.0001$.
