# Supplementary material for: Simultaneous augmentation of muscle and bone by locomomimetism through calcium-PGC-1α signaling
Source: Bone Res. 2022 Aug 3;10:52. doi: 10.1038/s41413-022-00225-w (PMC9345981; doi:10.1038/s41413-022-00225-w)
Supplement: Supplementary file 9 — Supplementary figure 9 [file 41413_2022_225_MOESM9_ESM.pdf]

Supplementary Fig. 9

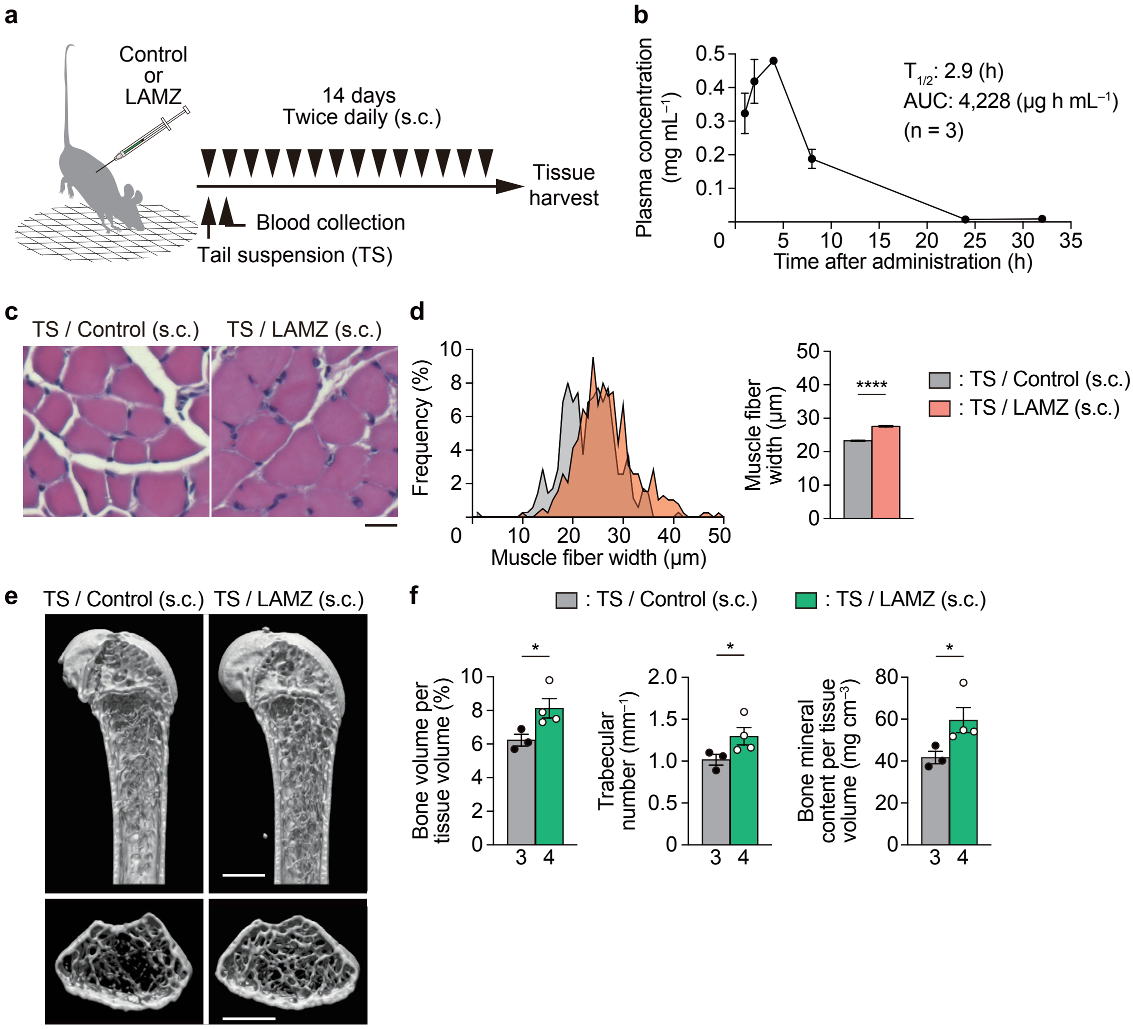

Supplementary Fig. 9 Subcutaneous injection of LAMZ improves both the muscle and bone of mice with locomotor frailty. (a) Schematic diagram of the experiment. **(b)** Plasma concentration of LAMZ after the subcutaneous injection. Peripheral blood was collected 1, 2, 4, 8, 24 and 32 h after the administration. **(c)** Representative histological images of the soleus muscle of the disuse model mice subcutaneously injected with LAMZ or a control solution. Cross sections of the muscle were stained with hematoxylin and eosin. Scale bar, 20 μm. **(d)** Distribution and mean width of the soleus muscle fibers. 3 and 4 mice in each group were analyzed. In total, the numbers of fibers measured were 350 and 388, respectively. **(e)** Representative micro-computed tomography (CT) images of the femur of disuse model mice subcutaneously injected with LAMZ or the control solution. Upper, sagittal section; and lower, transverse section of the metaphyseal area. Scale bar, 1 mm. **(f)** Bone parameters obtained by micro-CT analyses. Statistical analyses were carried out using Student's *t* test. The number of biological replicates is described below each bar. The error bars show the mean ± s.e.m. $*p < 0.05$; $****p < 0.0001$.
